# Genetic diversity analysis of big-bracted dogwood (*Cornus florida* and *C*. *kousa*) cultivars, interspecific hybrids, and wild-collected accessions using RADseq

**DOI:** 10.1371/journal.pone.0307326

**Published:** 2024-07-25

**Authors:** Erin L. P. Moreau, Ava N. Medberry, Josh A. Honig, Thomas J. Molnar

**Affiliations:** Department of Plant Biology, Rutgers University, New Brunswick, New Jersey, United States of America; Natural Resources Canada, CANADA

## Abstract

Big-bracted dogwoods are popular ornamental trees known for their beautiful spring blooms with showy bracts and four-season appeal. The two most widely grown species are *Cornus florida* and *Cornus kousa*, native to Eastern North America and East Asia. Despite their horticultural prominence, there is little information available regarding genetic diversity, population structure, relatedness, and subspecies origins of dogwood cultivars. In this study, 313 cultivars, wild-collected plants, and Rutgers University breeding selections, focusing on *C*. *florida*, *C*. *kousa*, and interspecific hybrids, were genotyped using restriction-site associated DNA sequencing (RADseq) generating thousands of single nucleotide polymorphism (SNP) and insertion deletion (Indel) markers. The research results showed high genetic diversity among *C*. *florida* and *C*. *kousa* wild-collected plants and cultivars. For *C*. *florida*, pink-bracted plants formed a distinct clade from those with white-bracts with the Mexican *C*. *florida* ssp. *urbiniana* forming an outgroup. For *C*. *kousa*, Chinese-collected plants (ssp. *chinensis*) were a distinct subspecies with clear separation from Japanese and Korean accessions (ssp. *kousa*) and cultivars were designated as ssp. *chinensis*, ssp. *kousa*, or ssp. hybrid. Using this information, a Kompetitive allele specific PCR (KASP) assay genotyping panel was designed to determine *C*. *kousa* trees’ subspecies makeup. Results revealed many cases of genetically identical cultivars being sold under different names, especially for pink-bracted cultivars of both species. Additionally, reported parent-progeny relationships were evaluated and either validated or discredited. Finally, the hybrid germplasm analysis validated pedigrees of interspecific F1 hybrids and found many of the recent Rutgers breeding selections contain small regions of pacific dogwood (*C*. *nuttallii*) DNA introgressed into *C*. *kousa* backgrounds. This diversity study elucidates origins, diversity, and relationships of a large population of big-bracted dogwoods. The results can inform plant breeders, arboreta, and the ornamental plant industry, as most modern cultivars and popular historic cultivars are represented.

## Introduction

Big-bracted dogwoods (Benthamidia clade of *Cornus* L.) are small ornamental trees prized for their beautiful spring blooms with showy bracts, striking red fruits, attractive fall color, and graceful form. The two most popular species are *Cornus florida* L. and *Cornus kousa* (Buerger ex. Miq) Hance [1]. *Cornus florida* is native to the eastern United States and blooms in early spring before its leaves are fully expanded. It is a culturally beloved harbinger of spring in the eastern United States and has been designated as the state flower or tree of Missouri, North Carolina, and Virginia. *Cornus kousa* blooms a month later than *C*. *florida* and is native to China, Korea, and Japan. In the past 30 years *C*. *kousa* has been gaining popularity in the United States due to its resistance to powdery mildew (PM) (*Erysiphe pulchra* Cook and Peck), dogwood anthracnose (*Discula destructiva* Redlin), and dogwood borer (*Synanthedon scitula* Harris), which are common problems for *C*. *florida* [2–5]. A third dogwood species from the pacific northwest of North America, *Cornus nuttallii* (Audubon ex Torr. & A. Gray), has a larger, more open growth habit with 4 to 6 bracts per inflorescence. However, *C*. *nuttallii* is rarely cultivated outside of its native range due to limited climate adaptability, and shares *C*. *florida*’s susceptibility to dogwood anthracnose and dogwood borer [1, 6]. The showy bracts of all three species are typically white, but cultivars with pink bracts are available for *C*. *florida* and *C*. *kousa*. Vigorous, disease-resistant hybrids between the three species have also been created and are popular in the nursery industry [1, 7]. Sales of dogwood trees are valued at 31 million dollars annually [8].

Breeding programs for big-bracted dogwoods are active at Rutgers University, the University of Tennessee, and North Carolina State University; however, most cultivars have been introduced by nursery professionals and hobby breeders through selection of sport mutations and trees grown from open-pollinated (OP) seed [1]. Once a superior plant is selected, it is propagated asexually, normally through chip budding. Cappiello and Shadow describe over 130 historical and current cultivars of both species [1]. Because of the lack of breeding data for most cultivars, there is limited information on their origins and how they are related which poses challenges for breeding. Further, there is evidence of possible mix-ups in the nursery trade as several cultivars are phenotypically and genetically similar but carry different names [9–11]. Knowledge of current genetic diversity and population structure of cultivars could help breeders more effectively maintain genetic diversity while breeding improved cultivars with combinations of desirable traits such as pink bracts and disease resistance. Maintaining sufficient genetic diversity through generations of phenotypic gains is a common plant breeding dilemma. It is particularly important for dogwoods as they exhibit inbreeding depression [12], similar to most self-incompatible, outcrossing plant species.

Recent diversity studies of wild-collected trees have shown population structure in *C*. *kousa* and *C*. *florida* based on geographic origin [13, 14]. Due to limited breeding, most cultivars are likely not very far genetically removed from wild-collected plants, and indeed some were derived directly from wild-collected seed. Provenance can have a considerable effect on the adaptability of woody plant germplasm with respect to abiotic stresses like drought and cold [15–17] and ornamental characteristics like fall color intensity [16]. In addition, there is confusion regarding the affiliation of many *C*. *kousa* cultivars to its two subspecies, ssp. *kousa* (Korean and Japanese origin) and ssp. *chinensis* (Osborn) Q. Y. Xiang (Chinese origin). Some authors consider *C*. *kousa* ssp. *chinensis* as the superior ornamental form of the species, describing increased vigor, earlier flowering, larger bracts, and excellent fall color [1, 18]. Clearly identifying the subspecies of each cultivar would allow for a better understanding of their genetic origins and an evaluation of whether ssp. *chinensis* cultivars are indeed horticulturally superior and could be marketed as such.

*Cornus florida* also has two subspecies but they are much more easily distinguished from one another. *Cornus florida* ssp. *florida* is the standard form of flowering dogwood with its characteristic fully expanded bracts and is widely distributed over most of *C*. *florida*’s native range [19]. *Cornus florida* ssp. *urbiniana* (Rose) Rickett has distinct narrow bracts that remain convergent at the apices, forming a “Chinese lantern” effect as well as fewer flowers and fruits per inflorescence [20]. It is native to Mexico, with the largest population found in the Sierra Madre Oriental mountains of Nuevo León State and small disjunct populations in the states of San Luis Potosí, Veracruz, and Tamaulipas [20, 21]. *Cornus florida* ssp. *urbiniana* is sold as a novelty by nurseries in the United States.

To date, intraspecific genetic diversity studies of big-bracted dogwoods have either focused on wild-collected accessions [13, 14, 22, 23] or cultivars and breeding program selections [9, 24–27]. Including both types of germplasm in a comprehensive study would provide valuable information on the wild origins of modern cultivars. Specifically studying wild-collected accessions in arboreta ex situ collections would also give insights into the state of ex situ conservation of big-bracted dogwoods. Ex situ collections serve as reservoirs of genetic diversity that often have valuable passport information and phenotypic evaluation. They constitute potential breeding pools more accessible than in situ trees, especially for species native to other continents.

Previous diversity studies with dogwood cultivars have used simple sequence repeat markers (SSRs), chloroplast DNA (cpDNA) haplotypes, or DNA amplification fingerprinting (DAF), a variation of Random Amplified Polymorphic DNA (RAPD) markers with shorter primers. The latter two methods are limited in their ability to yield ample intraspecific polymorphisms essential for population structure analysis and clonal accession determination. The restriction-site association DNA sequencing (RADseq) family of genotyping methods (also known as genotyping by sequencing, GBS) have become standard for diversity studies in species with limited genomic resources because of their ability to simultaneously develop and obtain thousands of single nucleotide polymorphisms (SNPs) and insertion deletion polymorphisms (Indels) in a cost effective manner without the need for a reference genome [28, 29]. In comparative studies in apple, *Arabidopsis halleri*, and Gunnison sage-grouse, large numbers of SNP markers were superior to SSRs in differentiating nuances in population structure [30–32]. Recently, RADseq has been used for genetic diversity studies of the ornamental plants *Eremurus spp*. [33], *Franklinia alatamaha* [34], *Hydrangea macrophylla* [35], *Papaver* spp. [36], *Rhododendron canescens* [37], and *Rhododendron meddianum* [38] as well as for wild-collected *C*. *florida* [14, 39] and in phylogeny studies of the *Cornus* genus [40]. Furthermore, large numbers of bi-allelic SNPs and Indels (n>300–1000) have been successfully used for parentage analysis [41–43], which was long considered best resolved by multi-allelic SSR markers. Therefore, the same set of SNP and Indel markers derived from RADseq can be used for both diversity evaluation and parentage analysis.

In this study, we used RADseq to discover SNPs and Indels in 313 cultivated and ex situ wild-collected big-bracted dogwoods and examine relationships, clonal identities, genetic diversity, and population structure. The research focuses on *Cornus florida*, *C*. *kousa*, known interspecific hybrids, and Rutgers breeding program selections with mixed, somewhat uncertain interspecific pedigrees. This is the first RADseq diversity study to include both cultivated and wild-collected *Cornus* germplasm for the primary purpose of understanding genetic relationships between and among cultivated germplasm in relation to wild *Cornus* sources.

## Methods

### Germplasm

**Fig 1:**
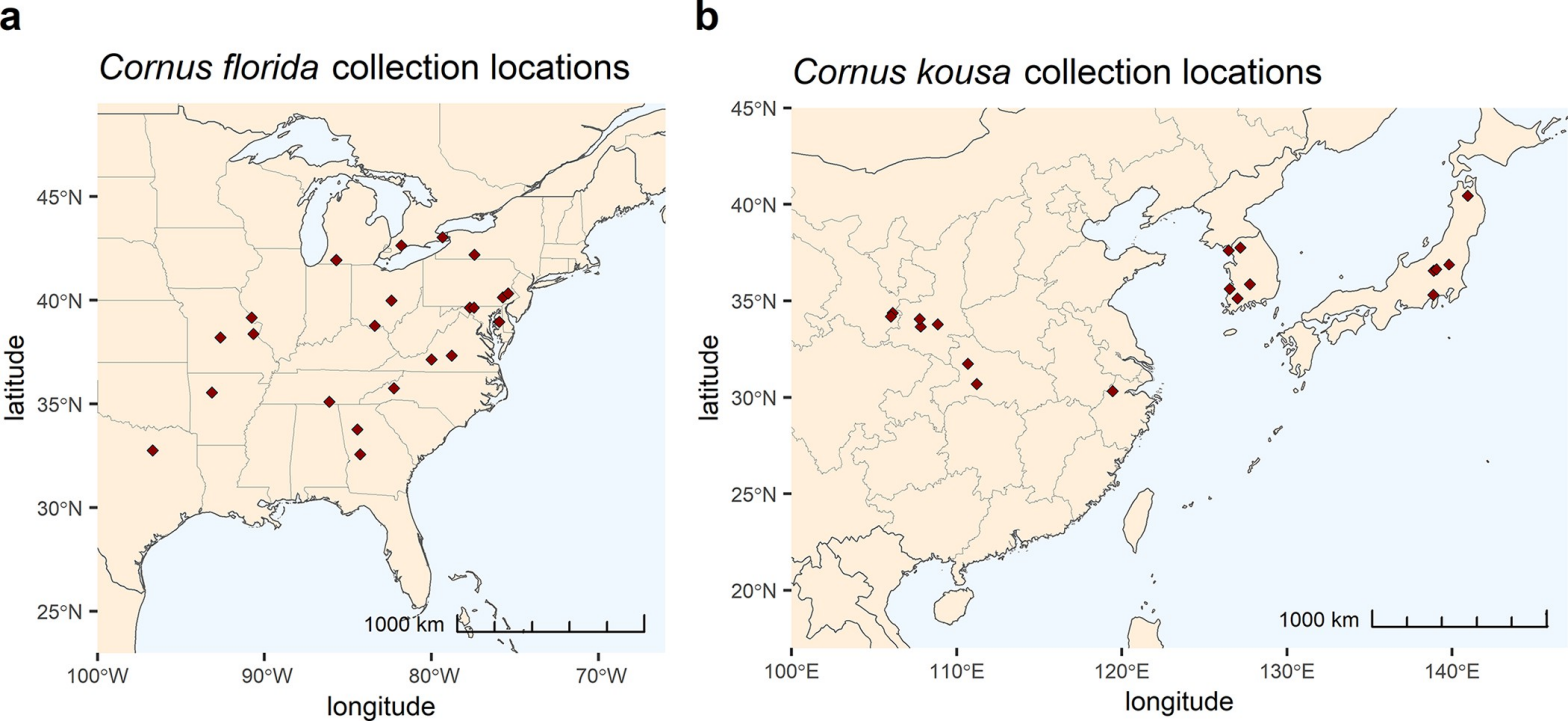
Geographic origins of wild-collected *Cornus* plants included in the genetic diversity study. a) *Cornus florida* collection locations in the United States and Canada. b) *Cornus kousa* collection locations in China, South Korea, and Japan. Made with Natural Earth. Free vector and raster map data @ naturalearthdata.com.

Cultivars and breeding selections were sampled from the Rutgers University dogwood breeding program (New Brunswick, NJ, USA) and arboreta, nursery, and private collections (S1 Table). Wild-collected plants were sampled from ex situ arboreta collections. Three trees were sampled per original collection location unless collection notes indicated that seed was collected from a single mother tree in which case only one tree was sampled. The original collection locations of wild-collected plants are shown in Fig 1, constructed with rnaturalearth using Natural Earth Data [44]. The 313 accessions were designated when received as 105 *C*. *florida*, 144 *C*. *kousa*, 53 interspecific hybrids, 7 *C*. *nuttallii*, and 4 outgroup species. Of the 313 accessions, 64 were of documented wild origin (including cultivars selected directly from the wild) while 249 were of garden (not collected directly from the wild) origin. The full list of accessions with relevant background information regarding institution sources, nursery sources, wild collection locations, and pedigree information can be found in S2 Table.

### DNA extraction and RADseq library preparation

Young leaves of all samples were flash frozen in liquid nitrogen or dried with silica gel and then ground with a Qiagen tissuelyser (Qiagen, Hilden, Germany). DNA was extracted using the DNeasy Plant kit (Qiagen, Hilden, Germany) with the following modifications: Tubes were kept frozen in liquid nitrogen until the addition of 500 μl AP1 buffer. The ice incubation was extended from 10 to 30 minutes and the subsequent centrifuge step was increased from 5 to 10 minutes. For the elution step, the AE buffer was heated to 35°C before addition to the columns and incubation was increased from 5 to 10 minutes.

The RADseq libraries were constructed using a protocol derived from Poland et al. [45]. Briefly, 200 ng DNA/sample was digested with the rare cutter PstI-HF and common cutter MspI (NEB, Ipswich, MA, USA) at 37°C for 2 h. For each sample, a uniquely barcoded forward PstI adapter and universal reverse MspI Y-adapter were ligated to the digested DNA in a master mix containing 2 μL of 10× NEBuffer 4, 200 U T4 DNA ligase, and 4 μL of 10 mM ATP per sample. The ligation was carried out at 22°C for 2 h, with a subsequent incubation of 65°C for 20 min to inactivate the ligase. Samples were cleaned using 0.5 v/v magnetic AMPure XP beads (Beckman Coulter, Indianapolis, IN, USA), with a 70% ethanol washing step to remove DNA fragments <300 bp. Individual cleaned library samples were subsequently PCR amplified with the following cycling parameters: initial denaturation at 95°C for 30 s; followed by 16 cycles of 95°C for 30 s, 62°C for 20 s, 68°C for 15 s; with a final extension of 68°C for 5 min. The PCR primers included sequences for Illumina (San Diego, CA, USA) flow cell binding. DNA libraries were quantified with the Qubit 3.0 flourometer (Thermo Fisher Scientific, Waltham, MA, USA), normalized to 9.0 ng/μL, and pooled into 48-plex sequencing libraries. A final cleanup step was performed with magnetic beads and ethanol wash as described previously. A total of seven pooled libraries were paired-end sequenced (2 × 150) on an Illumina HiSeq with 30% PhiX spike-in by GENEWIZ (South Plainfield, NJ, USA). After sequencing, FastQC was used to assess the quality of the raw sequencing data sets [46].

### Data analysis

#### All accessions

For all samples, single nucleotide polymorphic (SNP) marker and Indel discovery was carried out using the GBS SNP-Calling Reference Optional Pipeline (GBS-SNP-CROP) [47]. After the demultiplexing step, two demultiplexed files for *C*. *florida* H4AR15P28 and H4AR15P35 were added to the data set from a previous QTL mapping study using the same RADseq protocol [48]. Reads were aligned to the draft reference genome of *C*. *florida* ‘Appalachian Spring’ [49]. This initially returned 22,563 biallelic SNP markers and Indels for further analysis. Accessions *C*. *florida* 2016–084*B, *C*. *florida* ‘Little Princess’ USNA, and *C*. *nuttallii* ‘Goldspot’ were excluded from further analysis due to large amounts of missing data.

Further filtering and analysis of the original 22,563 biallelic SNPs and Indels was conducted to identify markers with a minor allele frequency (MAF) ≥ 0.05, missing data ≤ 0.10, and heterozygote frequency ≤ 0.90. A final filtering step was performed to choose one marker per 500 nucleotide region based on highest heterozygosity, resulting in 3009 markers for principal coordinates analysis (PCO) and Neighbor Joining (NJ) tree construction carried out in R [50]. A distance matrix was calculated using Gower’s dissimilarity index with the ‘vegan’ R package [51]. Principal coordinates analysis was conducted with the ‘ape’ R package [52]. When calculating the amount of variation explained by the principal coordinates, negative eigenvalues were replaced with zeros. The NJ tree was constructed and bootstrap (BS) analysis with 1000 bootstraps were performed with the ‘ape’ R package, and rooted to *C*. *capitata* ‘Mountain Moon’ [52] The reproducible R scripts and input files for all R analyses and data visualization can be accessed at https://github.com/pfarr019/DogwoodDiversity.

#### Separate analyses of *Cornus florida* and *Cornus kousa*

Based on the results of the initial analysis with all accessions, the non-admixed samples of *C*. *florida* and *C*. *kousa* (>0.90 membership to a single population) were split into separate groups for subsequent analyses. *Cornus kousa* 382–86*A was excluded from subsequent *C*. *kousa* analyses because it clustered with the *C*. *florida* accessions, indicating a mislabeled or misprocessed accession.

The groups were rerun separately through the GBS-SNP-CROP pipeline to find polymorphisms within each species group. For *C*. *florida*, reads were aligned to the *C*. *florida* reference genome. For *C*. *kousa*, reads were aligned to a mock reference constructed from sequencing data of *C*. *kousa* 1996–363*A, an accession with one of the largest demultiplexed RADseq fastq files. This initially returned 35,641 biallelic SNPs and Indels for *C*. *florida* and 42,481 for *C*. *kousa*. The SNPS and Indels were further filtered according to MAF, missing data, and heterozygosity as described above. For *C*. *florida*, only one marker (with the highest heterozygosity) was chosen per 500 nt region of the reference genome. For *C*. *kousa*, the marker with highest heterozygosity was chosen per GBS-SNP-CROP identified cluster. This resulted in 7,622 and 3,599 markers for downstream analysis in R for *C*. *florida* and *C*. *kousa*, respectively. Accessions with greater than 50% of missing marker data were excluded from further analysis: *C*. *florida* ‘Wonderberry’, *C*. *kousa* ‘Aget’, *C*. *kousa* ‘Baby Splash’, *C*. *kousa* ‘Moonlight’, and *C*. *kousa* 79163-L. Distance matrices were calculated with Gower’s dissimilarity and NJ trees were constructed as described above.

To quantify the similarity of accessions that grouped tightly in the NJ trees, a modified Gower’s coefficient of similarity (GS) [53] was calculated, ranging from 0 to 1 to quantify similarity based on bi-allelic SNPs and presence/absence of Indels.


$$S_{Gower}(x,y)=\frac{\sum_{i=1}^{m}s_{i}w_{i}}{\sum_{i=1}^{m}w_{i}}$$

Where *s*_*i*_ = 1 if the two genotypes are the same for a given marker (AA vs AA), 0.5 if the two genotypes differ by one allele (AA vs AB), and 0 if the genotypes differ by both alleles (AA vs BB). In addition, *w*_*i*_ = 1 if both accessions were genotyped for the marker in question and 0 if either is missing genotype data for the marker.

Genetically duplicate accessions (GS> 0.995) were removed before PCO, STRUCTURE, and k-means analysis. Duplicate accessions can affect these analyses by creating artificial population structure or overestimating the variation explained by existing population structure. To determine population structure, a nested Bayesian model-based clustering analysis was conducted using STRUCTURE 2.3.4 [54]. Marker sets were pruned to satisfy assumptions about markers in linkage equilibrium. To approximate genomic position for pruning, *Cornus kousa* denovo assembled clusters were aligned to the *C*. *florida* ‘Appalachian Spring’ reference genome using Burrows-Wheeler Aligner’s BWA-MEM and 94 markers in clusters that did not align were excluded from further analysis. Markers were pruned for LD using PLINK’s pairwise correlation with *r*^2^ = 0.5 and 100 marker sliding window resulting in 6,679 and 2,449 markers for *C*. *florida* and *C*. *kousa*, respectively [55]. Markers were further pruned in PLINK to 0.05 missing data or less and thinned randomly to 1000 markers due to STRUCTURE software analysis time and memory constraints. In STRUCTURE, all model-based clustering simulations were conducted using 100,000 burn-in Markov chain Monte Carlo (MCMC) iterations, followed by 100,000 MCMC run iterations for (*K*) = one through 10, with 10 replicates of each value of (*K*). Run simulations were setup to use an admixture ancestry model with correlated allele frequencies and tested under the assumption that all loci were independent and in linkage equilibrium. STRUCTURE HARVESTER was used to implement the Evanno deltaK method to determine optimal K values for populations [56, 57]. STRUCTURE results were visualized with ‘pophelper’ R package [58]. PCO analyses were performed for both species using R as described above for the analysis with all accessions. K-means clustering was executed with the ‘adegenet’ R package [59, 60]. Because K-means clustering is sensitive to missing data, only markers with 5% missing data or less were used (5065 total markers for *C*. *florida* and 1851 for *C*. *kousa*). All principal components were included in the analysis and the K-value with the lowest Bayesian Information Criterion (BIC) were chosen as the optimal value.

Parent-progeny relationships between accessions were investigated. To decrease the search space, wild-collected accessions were excluded from the dataset unless they were suspected to be the result of hybridization of cultivated germplasm or used as breeding parents. The non-duplicate accessions were assigned one of three genotype classes for analysis based on historical data: parent, offspring, or all (analyzed as both a possible parent and offspring). The R package ‘apparent’ [41] was used to identify triads (offspring and parents when both parents are sampled) for α < 0.05. For Dyads (offspring and one parent when one parent is unsampled) a similar approach using a pair of Dixon tests was adopted for α < 0.05 [61, 62]. Based on the approach of Hayes [42], for all possible dyad combinations, the proportion of loci that were in opposite homozygous states (*P*_*OHL*_) was calculated with the following formula:

$$P_{OHL}(x,y)=\frac{\sum_{i=1}^{m}o_{i}w_{i}}{\sum_{i=1}^{m}w_{i}}$$

Where *o*_*i*_ = 1 if the two genotypes are in opposite homozygous states (AA vs BB) and *o*_*i*_ = 0 if the genotypes have at least one allele in common (ie AA vs AB). In addition, *w*_*i*_ = 1 if both accessions were genotyped for the marker in question and 0 if either is missing genotype data for the marker. Theoretically, a parent and progeny pair will not have any loci where the parent and the progeny are opposite homozygotes (*P*_*OHL*_ = 0), however, a small amount of error will occur with Illumina sequencing. After ordering the dyads according to *P*_*OHL*_, the size of gaps between ordered dyad’s *P*_*OHL*_ values were calculated. The first Dixon test was applied to determine if the largest gap in the first half of the ordered samples was an outlier compared to 29 other randomly sampled gaps. If the gap’s size was significant (an outlier), then Dixon tests were used to test the individual significance of each dyad below the gap compared to a sample of the 29 lowest dyads above the gap (spurious dyads).

For *C*. *kousa*, an analysis of molecular variance (AMOVA) was performed with R package ‘poppr’ to detect population differentiation between the two subspecies [63]. For the AMOVA, the non-duplicate *C*. *kousa* accessions used in the PCO and STRUCTURE analyses were further filtered to remove individuals with <90% membership to one population according to the STRUCTURE results (cultivars with admixture between ssp. *kousa* and ssp. *chinensis*), to 76 accessions. Poppr’s default parameters of using only markers with ≤5% missing data were applied, resulting in 1,951 markers used for this analysis. An AMOVA was not performed for the *C*. *florida* subspecies because there were only a few representatives of *C*. *florida* ssp. *urbiniana* in the dataset.

#### KASP genotyping panel development

A Kompetitive allele specific PCR (KASP) marker genotyping panel was designed to distinguish between *C*. *kousa* ssp. *kousa*, ssp. *chinensis*, and ssp. hybrids. Fifteen SNPs or Indels were chosen for KASP assay development that were fixed differences between the subspecies and had centralized locations in Illumina mockreference cluster sequences with sufficient flanking sequences for primer design. Three primers were designed for each of these polymorphisms: a universal reverse primer and two forward primers that only differed in one nucleotide, the polymorphism, at the 3’ end. For each primer set, the sequence complimentary to the FAM fluorophore quencher (GAAGGTGACCAAGTTCATGCT) was appended to the 5’ of the ssp. *chinensis* forward primer (1) and the sequence complimentary to the HEX fluorophore quencher (GAAGGTCGGAGTCAACGGATT) was appended to the 5’ of the ssp. *kousa* forward primer (2). Primer sequences and summary statistics are available in S3 Table. Primers were synthesized by Integrated DNA Technologies (Coralville, USA).

A primer master mix was made for each KASP genotyping assay with the following components: 18 μl 100 μM forward primer 1, 18 μl 100 μM forward primer 2, 45 μl 100 μM reverse primer, and 69 μl H_2_O. Then for each KASP assay, a KASP master mix was assembled with 11.9 μl of the appropriate primer master mix and 432 μl LGC Genomics master mix (LGC Group, Teddington, UK) containing universal FRET cassettes (FAM and HEX), ROX reference day, Taq polymerase, nucleotides, and MgCl_2_.

KASP reactions were assembled in 96 well plates with 4 μl KASP master mix and either 4 μl genomic DNA from individual plants (5 ng/μl) or 4 μl H_2_O for the non-template controls. The assays were run on an Applied Biosystems StepOnePlus Real-Time PCR System (Thermo Fisher Scientific, Waltham, US). The following cycling conditions were used: 30°C for 1 min (initial fluorescence recorded), then 94°C for 15 min, followed by 10 touchdown cycles of 94°C for 20 sec and 1 min of 61°C decreasing by 0.6°C each cycle to 55°C, 18 cycles of 94°C for 20 sec and 55°C for 1 min, followed by 8 cycles of 15 sec 95°C, 1 min 60°C, and 30 sec 23°C (fluorescence recorded), and a final fluorescence recording stage at 30°C for 1 min. ROX dye was used as the passive reference. The HEX fluorescence was read by the machine as VIC, as the StepOnePlus does not have programming to read HEX and the two dyes have similar excitation and emission spectra.

Initially, the fifteen KASP assays were tested on positive controls for *C*. *kousa* ssp. *chinensis* (‘Greensleeves’ and ‘Wolfeyes’), ssp. *kousa* (‘Satomi’ and ‘Benifuji’), and ssp. hybrids (‘Moonbeam’ and ‘National’). Seven KASP assays sufficiently amplified the FAM and HEX signals to allow for allelic discrimination between homozygotes for allele 1 (ssp. *chinensis*), allele 2 (ssp. *kousa*), or heterozygotes (hybrids). These seven assays were tested on unknown samples, positive controls in duplicates, and three non-template controls. Clustering and marker assignment were performed with the StepOne software’s allelic discrimination function. For each marker, ssp. *chinensis* homozygotes were assigned a value of 0, ssp. *kousa* homozygotes were assigned a value of 1, and heterozygotes were 0.5. The mean value of the KASP genotyping panel assays were compared to the mean of the same markers for the RADseq data and the STRUCTURE analysis from the top 1000 RADseq markers.

#### Hybrid analysis

To examine the interspecific hybrid composition of Rutgers breeding selections where multiple generations of crossing and intercrossing have occurred, known and suspected interspecific hybrids and presumed *C*. *kousa* Rutgers breeding selections were analyzed alongside reference accessions of each species—10 *C*. *florida*, 10 *C*. *kousa*, and 6 *C*. *nuttallii*. For *C*. *florida* and *C*. *nuttallii*, species references were chosen that had large demultiplexed sequencing files and were spread across the neighbor joining (NJ) tree to represent the genetic diversity of each species. Only six pure *C*. *nuttallii* were available for use as species controls because one accession, 2021 RC, unexpectedly presented as a hybrid in the initial analysis with all accessions.

The analysis was rerun through the GBS-SNP-CROP pipeline with alignment to the *C*. *florida* reference genome, initially returning 29,544 biallelic SNP markers and Indels. Strict further filtering was conducted to identify species specific markers based on the reference accessions. Markers were filtered using the following criteria: present in ≥ 88% of the reference accessions, homozygous in reference accessions, not polymorphic within each reference group (only homozygous for one state ─ AA or BB), and polymorphic between reference groups. This yielded 2889 species specific *C*. *florida* markers, 2893 *C*. *kousa* markers, and 2703 *C*. *nuttallii* markers. To equalize the number of species-specific markers for all three species, *C*. *florida* and *C*. *kousa* markers were further filtered down to 2703 markers each for a total of 8109 markers. This was done by only keeping a maximum of three markers per 500 bp *C*. *florida* genomic region based on highest percentage of individuals genotyped, in addition to deleting about 100 of the remaining markers with the lowest percentage of individuals genotyped. The percentage of each species’ markers in the genome was calculated for each accession with the following equations:

$$Perc(Florida)=\frac{\sum {LF}_{i}}{\sum {LF}_{i}+{LK}_{i}+{LN}_{i}}X\;100$$

$$Perc(Kousa)=\frac{\sum {LK}_{i}}{\sum {LF}_{i}+{LK}_{i}+{LN}_{i}}X\;100$$

$$Perc(Nuttallii)=\frac{\sum {LN}_{i}}{\sum {LF}_{i}+{LK}_{i}+{LN}_{i}}X\;100$$

For each species-specific locus *L*(*F*, *K*, *or N*)_*i*_, *L*_*i*_ = 1 when homozygous for the species-specific allele, *L*_*i*_ = 0.5 when heterozygous for the species-specific allele, and *L*_*i*_ = 0 when homozygous for the non-species-specific allele or missing data. Additionally, PCO analysis was conducted with the 8109 markers as previously described.

Pedigree records were used to calculate expected percentage of species makeup for each Rutgers *C*. *kousa* breeding program accession for comparison to calculated species makeup. Based on field notes documented by Elwin Orton (E. Orton, unpublished) open pollination (OP) events were assumed to be crosses with *C*. *kousa* or other interspecific breeding selections with significant *C*. *kousa* origins. Thus, *C*. *kousa* was considered the majority contributor to calculate expected percentages during those events. For example, a *C*. *kousa* × *C*. *florida* F1 hybrid is expected to be 50% of each species. When backcrossed to *C*. *kousa*, the amount of *C*. *florida* DNA is expected to decrease approximately by half, so the expected species makeup is 75% *C*. *kousa* and 25% *C*. *florida*. If the next generation’s pedigree shows an OP event, it is assumed to be a cross with *C*. *kousa*, therefore the percentage of *C*. *florida* DNA is expected to decrease by half again, approximating 87.5% *C*. *kousa* and 12.5% *C*. *florida*.

## Results and discussion

### Sequencing data statistics

The raw paired end fastq files for the seven sequencing sets contained 551.7 Gb of raw data. The mean Q values for each sequencing set ranged from 34.39 to 37.86 with an overall average of 36.29. The average number of paired end reads per accession for demultiplexed files was 1.25 million, with a standard deviation of 0.54 million reads.

### All accessions

**Fig 2:**
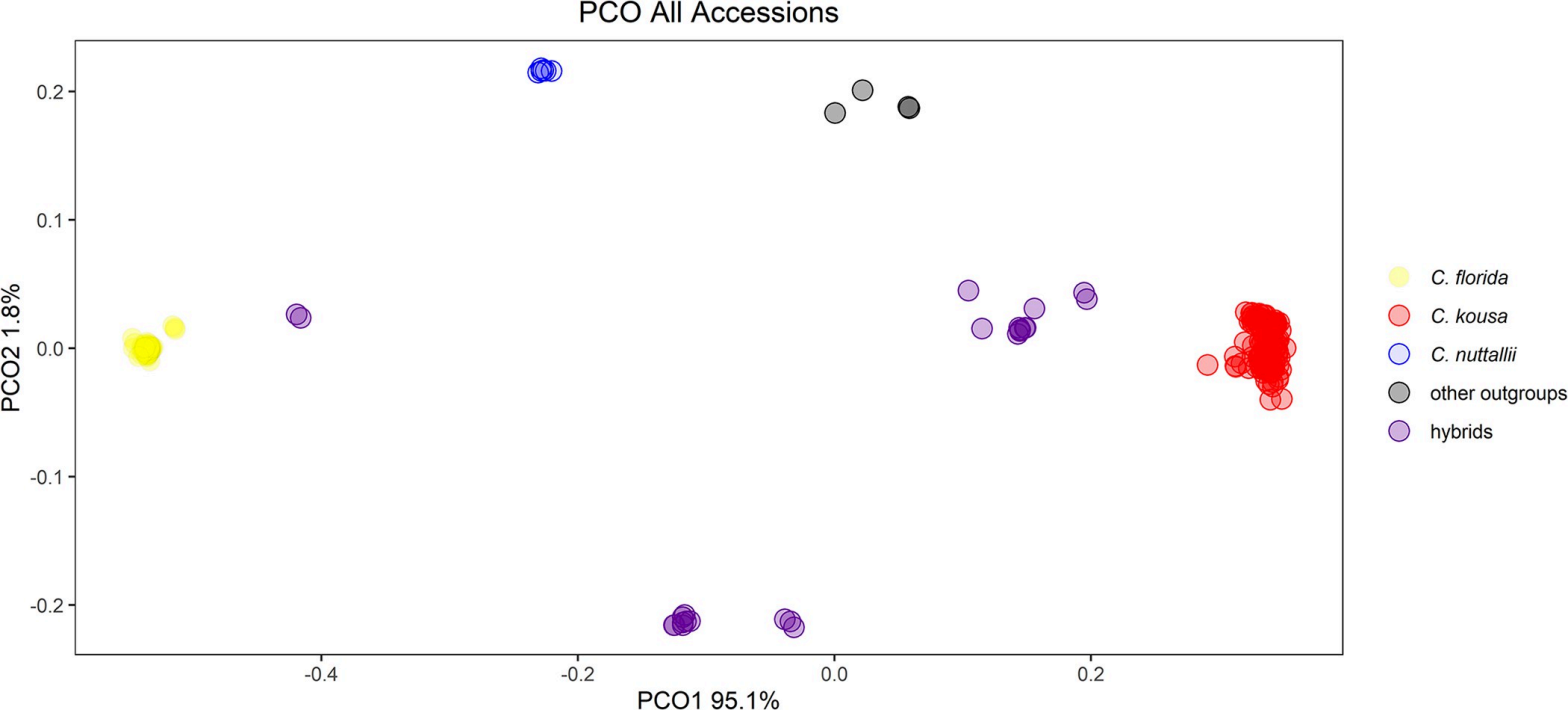
Principal coordinate analysis (PCO) of 313 big-bracted dogwood (*Cornus*) cultivars, breeding selections, and wild-collected accessions with 3009 RADseq derived markers.

*Cornus florida* and *C*. *kousa* accessions were clearly separated by the initial PCO analysis. The first principal coordinate (PC) explained 95.1% of the variation and distinguished *C*. *florida* from *C*. *kousa*, with the hybrids and outgroups falling intermediate (Fig 2A). The second PC only explained 1.8% of the variation but distinguished the different hybrid groups from each other and the outgroup species. The large differences between *C*. *florida* and *C*. *kousa* obscured variation within the two species in the overall NJ tree (S1 Fig.), necessitating a rerun of the GBS-SNP-CROP pipeline separately for each species.

### Cornus florida

**Fig 3:**
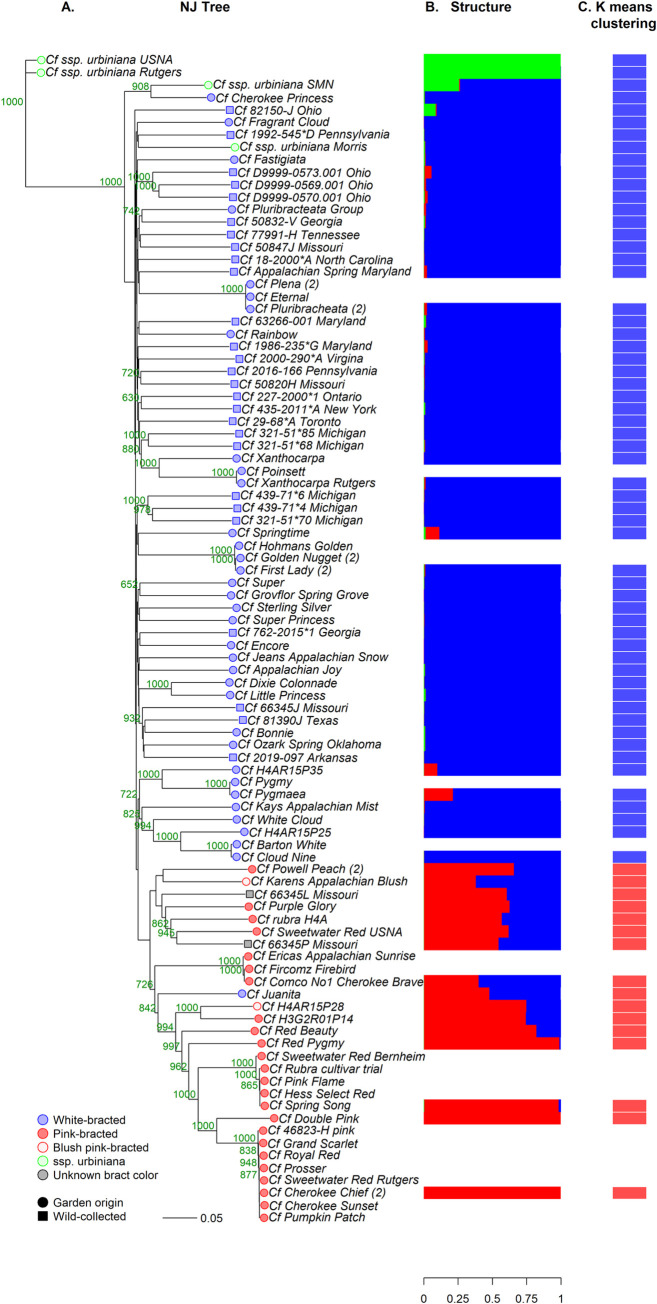
Genetic grouping of 92 *Cornus florida* dogwood cultivars, breeding selections, and wild-collected accessions with RADseq derived markers. A) Neighbor joining (NJ) tree from the Gower’s dissimilarity matrix of 7,622 single-nucleotide polymorphisms (SNPs) and insertion deletion polymorphisms (Indels). Bootstrap values are indicated in green. Wild-collected accessions’ names are followed by state or province of origin. Cultivars sampled twice from different institutions and with clonal similarity are followed by (2). Cultivars that were sampled twice from different sources and were not clonally identical are followed by the name of the source. B) Population structure for genetically unique accessions using mixed-model analysis of top 1000 SNPs and Indels using STRUCTURE software. C) k-means clustering results for genetically unique accessions with 5,065 markers (5% or less missing data).

**Fig 4:**
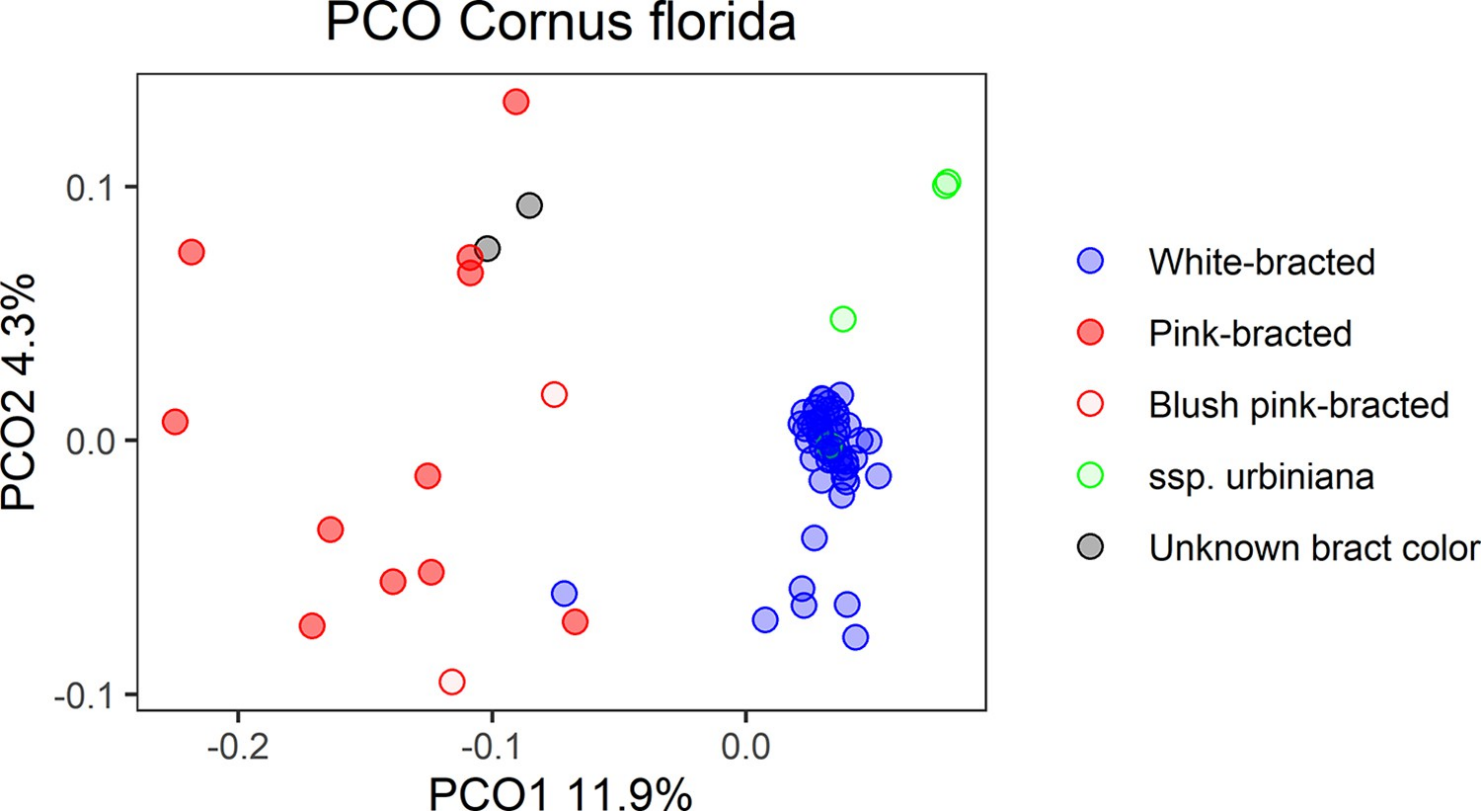
*Cornus florida* principal coordinate analysis (PCO). The analysis was performed with the same non-duplicate accessions as the STRUCTURE and k-means analyses.

For *Cornus florida*, the analysis separated the accessions into three main groups: ssp. *urbiniana*, pink-bracted ssp. *florida*, and white-bracted ssp. *florida* (Figs 3 and 4). In addition, there were many accessions that were genetically identical, especially for pink-bracted spp. *florida*.

The ssp. *urbiniana* (Mexican origin) accessions were distinct from the main group of ssp. *florida* accessions. However, only two of the four labeled ssp. *urbiniana* were pure ssp. *urbiniana* based on the NJ, STRUCTURE, and PCO analysis. The USNA and Rutgers’ accessions were ssp. *urbiniana*, the Morris accession was spp. *florida*, and the SMN accession appeared to be an F_1_ subspecies hybrid. The ssp. *urbiniana* group was not designated by the k-means for k = 2, but was for k = 3, which had a slightly higher BIC value. If a greater number of pure ssp. *urbiniana* accessions had been included, this group may have been identified in the k-means clustering analysis as well. In the PCO analysis, the third principal coordinate (4.1% of the variation, not shown) separated the ssp. *urbiniana* accessions from the ssp. *florida* with the hybrid ssp. *urbiniana* SMN falling intermediate. Further investigation into the origins of ssp. *urbiniana* SMN revealed that it is an open-pollinated progeny from a wild-collected ssp. *urbiniana* (Horsetail falls, Mexico) grown at the Stephen F. Austin University Gardens. According to our results, it is the result of hybridization with a ssp. *florida* tree. The presence of this hybrid accession in the NJ tree lowered the bootstrap value of the pink-bracted group because it was genetically similar to ‘Cherokee Princess’ and ‘Karen’s Appalachian Blush’ as well as the pure ssp. *urbiniana* and jumped between the two groupings during bootstrapping. When it was withheld from the analysis the bootstrap values of the pink group increased from 272 to 997 (S2 Fig).

All blush pink- and pink-bracted cultivars and breeding selections grouped together in the NJ diagram (Fig 3A). This group was also supported by the STRUCTURE results, with all pink- or blush pink-bracted individuals having at least 40% membership in the red population (Fig 3B). The k-means analysis determined blush-pink or pink bracted individuals as belonging to population 2 (Fig 3C). In the PCO analysis, the first principal coordinate, explaining 11.9% of the variation, separated the pink and blush-pink accessions from the white-bracted (Fig 4). The only documented white-bracted accession in this pink-bracted group was ‘Juanita’, a selection from Paul Cappiello with large bracts and unknown pedigree. Two wild-collected accessions from Elsberry Missouri, 66345L and 66345P, also grouped with the pink-bracted individuals. The bract color of these trees was never documented because they were weak trees planted in a remote location of the US National Arboretum. They did not survive the winter after their leaf tissue was sampled. Interestingly, they did not group with the other individual collected from the same location, 66345J, which was documented to have white bracts with pink clefts. It is possible that these two wild-collected accessions were also pink or blush-pink bracted based on the NJ tree, STRUCTURE, k-means, and PCO analysis results.

Pink-bracted *Cornus florida* are often referred to as forma *rubra*. Normally, individuals within a forma share a noticeable morphological deviation from the species but are not necessarily closely related. In the case of *C*. *florida*, f. *rubra* may designate a clade. *Cornus florida* f. *rubra* individuals are rare but geographically widespread in the wild. *Cornus florida* f. *rubra* was first documented in Virginia and introduced to horticulture by the English naturalist Mark Catesby in the early 1700’s [18, 64]. A search of the SEINet North American plant network and Smithsonian Institution’s online herbarium records revealed 13 wild-collected f. *rubra* specimens out of a total of 11,849 *C*. *florida* records. The oldest record dates from 1888 and f. *rubra* has been documented in Arkansas, Missouri, Pennsylvania, Virginia. It is unknown if the rare f. *rubra* wild accessions would also cluster with the f. rubra cultivars in the NJ tree or if the observed f. *rubra* cultivated clade is due to a genetic bottleneck caused by few founders and high relatedness.

There were several groupings of pink-bracted accessions that were genetically identical (Gower’s similarity (GS) ≥ 0.995) (Fig 3A). In some cases, these were variegated sports grouping with the cultivars that they were derived from. This is expected as bud sport mutants are typically genetically identical to the original plant except for the new mutation [65]. *C*. *florida* ‘Fircomz’ Firebird® and ’Comco No. 1’ Cherokee Brave™ shared a GS of 0.999 and *C*. *florida* ‘Sunset’ Cherokee Sunset™ was nearly identical to *C*. *florida* ‘Cherokee Chief’ with a GS of 0.999 [66]. Additional cultivars that were not expected to share GS greater than 0.995 were found. ‘Pumpkin Patch’ is listed in Cappiello and Shadow as a seedling selection [1], however, it was genetically identical to ‘Cherokee Chief’. Phenotypically, the two cultivars are distinct, as ‘Pumpkin Patch’ has interesting two-toned leaves with orange spring and fall color, is more susceptible to PM, and is less floriferous. Another seedling selection that shared a high GS value with its mother plant is ‘Erica’s Appalachian Sunrise’. This recent release from the University of Tennessee was documented to be a cross of ‘Cherokee Brave’ and ‘Karen’s Appalachian Blush’. For the patent application, SSR genotyping with 9 loci was performed for the trio [67]. ‘Erica’s Appalachian Sunrise’ had the same SSR profile as ‘Cherokee Brave’, therefore the authors concluded it was a self of ‘Cherokee Brave’. In this study the two plants had a GS of 0.998. ‘Erica’s Appalachian Sunrise’ is phenotypically distinct from ‘Cherokee Brave’ with a stronger central leader, increased powdery mildew resistance, and 5% of its inflorescences displaying darker red bracts [67].

These two cases raise the possibility of rare apomixis occurring in *C*. *florida*, which has never before been documented. *Cornus florida* exhibits sporophytic self-incompatibility [68]. If, in the case of ’Erica’s Appalachian Sunrise’, selfing barriers were overcome resulting in a "self" of ’Cherokee Brave’, it is expected that each heterozygous locus would have a 50% chance of becoming homozygous in the next generation. With thousands of heterozygous loci, one would then expect a more dissimilar GS value than 0.995, for example the theoretical GS of a ‘Cherokee Brave’ "self" with the current study’s data is 0.922. Therefore, ‘Erica’s Appalachian Sunrise’ presents the possibility that it is an apomictic progeny of ‘Cherokee Brave’ rather than the product of self-pollination. However, apomixis has never been experimentally confirmed in the genus *Cornus* and would have to be investigated further.

The possibility of apomixis events makes interpretation of the following results more difficult, as there are many unexpectedly genetically identical f. *rubra* cultivars. It is difficult to determine which could be the result of apomixis, mix-ups in collections or renamings in the trade, as f. *rubra* cultivars are highly desirable. Interestingly, in both cases of potential apomixis noted in the previous paragraph, the progeny are phenotypically distinct from the parents.

Other cultivars in the ‘Cherokee Chief’ clonal group along with ‘Cherokee Sunset’ and ‘Pumpkin Patch’ are 46823-H, ‘Grand Scarlet’, ‘Royal Red’, and ‘Prosser’ (Fig 3A). Although no longer widely sold, ‘Cherokee Chief’ and ‘Sweetwater Red’ were two of the most popular pink-bracted cultivars in the second half of the 20^th^ century. The ‘Sweetwater Red’ cultivar seems to be thoroughly confused, with different clones from three different sources genetically distinct, one of which groups in the ‘Cherokee Chief’ group, and another in the ‘Spring Song’ group. Although genetically distinct, the USNA and Bernheim clones were both sourced directly from Boyd Nursery which introduced the cultivar [1].

In addition to these pink-bracted cultivars, there are several other phenotypically indistinguishable cultivars in this diversity study that have GS of > 0.995 and are likely clones of each other which had been previously documented or suspected (Fig 3) [11, 69]. These included ‘Cloud Nine’ and ‘Barton’s White’ (synonym ‘Barton’); the sterile, double bracted ‘Pluribracheata’, ‘Plena’, and ‘Eternal Dogwood’; and variegated cultivars ‘First Lady’, ‘Golden Nugget’ and ‘Hohman’s Golden’.

*Cornus florida* f. *xanthocarpa* is a designation for rare, yellow-fruited individuals [70]. The three *xanthocarpa* trees in the study formed a distinct group with high bootstrap support (Fig 3A). Additionally, the unlabeled f. *xanthocarpa* tree at Rutgers Gardens was identified as ‘Poinsett’.

In the NJ tree (Fig 3A), there was a grouping (BS = 932) of three of the south-western collected plants (origins Missouri, Texas, and Arkansas) along with ‘Bonnie’, a cultivar introduced by Louisiana State University and ‘Ozark Spring’ from seed collected in the Crookson Hills region of Oklahoma (Fig 3A) [1]. This southwestern group corresponded geographically with the K = 2 western population observed in a more comprehensive RADseq diversity study of wild-collected *C*. *florida* [14]. However, previous genetic diversity studies focusing on wild-collected *C*. *florida* have showed only subtle population structure, likely due to long range seed dispersal [14, 22, 23]. Indeed, the current study did not detect strong population structure in wild-collected ssp. *florida* as the three STRUCTURE designated populations corresponded to f. *rubra* and the two main subspecies (ssp. *florida* and ssp. *urbiniana*).

This study focused on ex situ, wild-collected *C*. *florida* from arboreta and botanic gardens’ collections, so temporal and spatial sampling of wild plants was not rigorous. However the results provide insight into the state of ex situ conservation of *C*. *florida*. The collections of the five arboreta sampled showed a bias towards collection at the outskirts of the species native range (Fig 1A), where unique genetics may help these individuals thrive. Despite a 49% population decline of *C*. *florida* over its native range due to dogwood anthracnose and other factors [71], considerable genetic diversity still exists in wild populations due to large effective population sizes [14, 23, 72]. Therefore, ex situ genetic conservation of *C*. *florida* is currently not a priority of arboreta. In the present study, similar levels of genetic diversity and heterozygosity was observed in both *C*. *florida* wild-collected plants and white-bracted cultivars, evidenced by long terminal branch lengths in the NJ tree and lack of population structure.

Cultivars with documented powdery mildew (PM) resistance and tolerance are spread out in the NJ tree, showing the genetic diversity of PM-resistant accessions (Fig 3A). These include ‘Appalachian Joy’, ‘Jean’s Appalachian Snow’, ‘Kay’s Appalachian Mist, ‘Karen’s Appalachian Blush’, and Rutgers breeding selection H4AR15P25. This genetic diversity may translate into different genetic mechanisms of PM resistance. PM resistance Quantitative Trait Loci (QTL) studies have been limited so far, but resistance QTL were found on Chromosome 3 for H4AR15P25 [48] and on Chromosomes 1, 5, and 9 in two TN breeding program selections (not sampled) [73]. Genetic diversity in the accessions is not surprising as many of the Appalachian series cultivars were selected from wild-collected seeds sourced from Tennessee, Georgia, and Alabama [74]. Additionally, a cultivar with limited information, ‘Super Princess’, has been observed to be PM resistant at the Morris Arboretum and merits further evaluation in different environments. The genetic diversity of accessions with PM resistance is more promising for breeding than that of f. *rubra* accessions.

#### Parentage analysis

Although multiallelic SSRs are the most commonly used markers for parentage analysis, newly developed tools have enabled parentage analysis with large numbers (>1000) of biallelic SNPs and Indel markers. For apparent’s triad analysis, for each possible parental pair, an expected progeny genotype (EP) is constructed using only markers that are homozygous in both parents [41]. The EP genotype is compared to each potential offspring’s genotype using Gower’s Dissimilarity (GD) genetic distance. Theoretically, a true progeny should not have any genotypic mismatches with the expected progeny (GD = 0), but with a small amount of sequencing and genotyping error inherent in Illumina sequencing a small proportion of mismatches will occur and the GD will be greater than zero. Even so, the GD will still be smaller than the GD between EP and all false offspring. The significant gap between false and true triads’ GD values can be identified and used to assign significance to true triads with a pair of Dixon tests (S3A Fig). Apparent’s triad analysis identified ‘Double Pink’ as the progeny of ‘Cherokee Chief’ and ‘Spring Song’ (or one of their associated clones) with p = 0.0000 and GD of 0.003. Then, a similar approach was followed to identify dyads, parent-progeny pairs when one parent is missing from the data set. The proportion of loci with opposite heterozygous states (POHL) was calculated for each pair of accessions. A true dyad pair should have a POHL = 0, but with a small amount of sequencing and genotyping error inherent in Illumina sequencing a small proportion of mismatches will occur and the POHL value will be greater than zero. The significant gap between false and true dyad’s POHL values was identified and used to assign significance with a pair of Dixon tests. For each significant dyad, historical information was used to determine which cultivar was older and therefore the parent. The dyad analysis identified 15 significant dyads (p<0.05) plotted in red in S2 Fig. H4AR15P28 was assigned as the parent of H3G2R01P14 (p<2.2E-16, *P*_*OHL*_ = 0.003), which conforms with pedigree data from the Rutgers breeding program. Additionally, both ‘Cherokee Chief’, and ‘Spring Song’ were identified separately as the parents of ‘Double Pink’ (p<2.2E-16, *P*_*OHL*_ = 0.003 & (p<2.2E-16, *P*_*OHL*_ = 0.004), which agrees with apparent’s triad analysis. Together, these results serve to validate the dyad analysis method. Furthermore, ‘Cherokee Chief’ was assigned as the parent of several pink or blush accessions, including ‘Purple Glory’ (p<2.2E-16, *P*_*OHL*_ = 0.006), ‘Powell Peach’ (p<2.2E-16, *P*_*OHL*_ = 0.007), ‘Sweetwater Red’ from USNA (p<2.2E-16, *P*_*OHL*_ = 0.007), the f. *rubra* accession from Rutgers field H4A (p<2.2E-16, *P*_*OHL*_ = 0.007), 66345L (p<2.2E-16, *P*_*OHL*_ = 0.007), 66345P (p<2.2E-16, *P*_*OHL*_ = 0.006), and ‘Spring Song’ (p<2.2E-16, *P*_*OHL*_ = 0.007). The parent-progeny relationship of ‘Cherokee Chief’ and ‘Spring Song’ is unexpected as they are both the parents of ‘Double Pink’, however this possible inbreeding may explain ‘Double Pink’s sterile inflorescences with double bracts and extreme powdery mildew susceptibility. 66345L and 66345P are both wild-collected accessions from Missouri, so it appears they are the product of pollination from a nearby ‘Cherokee Chief’, which has been widely sold and planted since it was released in the 1950s. Therefore, truly wild-origin pink-bracted trees may be genetically distinct. ‘Cherokee Princess’ was the parent of the hybrid ssp. *urbiniana* SMN, (p< 2.2E-16, *P*_*OHL*_ = 0.005) and of ‘Karen’s Appalachian Blush’ (p<2.2E-16, *P*_*OHL*_ = 0.008), explaining why it was jumping between the two accessions during bootstrapping. ‘Spring Song’ was determined to be the parent of ‘Juanita’ (p<2.2E-16, *P*_*OHL*_ = 0.006), accounting for ‘Juanita’s’ position as the sole white-bracted tree in the pink-bracted group. ‘Little Princess’ and ‘Dixie Colonnade’ share a dyad relationship (p<2.2E-16, *P*_*OHL*_ = 0.006), although it is not clear which one is the parent. The two ssp. *urbiniana* accessions also showed up as a significant dyad (p = 0.006, *P*_*OHL*_ = 0.012), however, these results may be a product of the analysis. Due to the small ssp. *urbiniana* sample size, ssp. *urbiniana* private alleles that may exist in opposite homozygous states between the two accessions would not have passed the minor allele frequency filter. Therefore, even though the *P*_*OHL*_ was a significant outlier according to the Dixon test, the results for this dyad should be interpreted with caution.

The dyad analysis plot shows a gap before 95 dyads with *P*_*OHL*_> 0.15, the highest values in the *C*. *florida* analysis. These dyads all include one of the two pure ssp. *urbiniana*, demonstrating how genetically divergent they are from the rest of the ssp. *florida* accessions. A similar uneven bimodal distribution of *P*_*OHL*_ values was seen in another dataset with an unequal number of representatives from two genetic groups [42].

The pink-bracted accessions in the study are overrepresented in the parentage results (‘Cherokee Chief’, ‘Spring Song’, ‘Double Pink’, ‘Powell Peach’, ‘Purple Glory’, USNA’s ‘Sweetwater Red’, H4A rubra, H4AR15P28, and H3G2R01P14) showing their high interrelatedness. ‘Cherokee Chief’ has been a particularly important breeding parent of pink-bracted accessions. These insights into relatedness will be used to inform breeding crosses to minimize inbreeding in f. *rubra*.

In other studies with similar tools for determining dyad parentage relationships, 0.010 is a generally accepted threshold for POHL values between true and spurious dyads [43, 75]. This was quite close to the experimental values seen for the *C*. *florida* data. The advantage of using the Dixon tests is that significance values can be assigned to the dyads and the threshold is based on experimental data, as the level of error may vary slightly depending on the dataset.

### Cornus kousa

For *Cornus kousa*, the analysis separated accessions into two main subgroups, ssp. *chinensis* and ssp. *kousa*, with genetically intermediate subspecies hybrids. The two subspecies groups were validated by the inclusion of wild-collected materials, but also included cultivars and Rutgers breeding selections. The hybrid accessions consisted exclusively of non-wild accessions which reflects the admixture that occurs in breeding, especially in the Rutgers dogwood breeding program. There was also significant cultivar synonymy within the pink bracted and ssp. *chinensis* cultivar groups.

**Fig 5:**
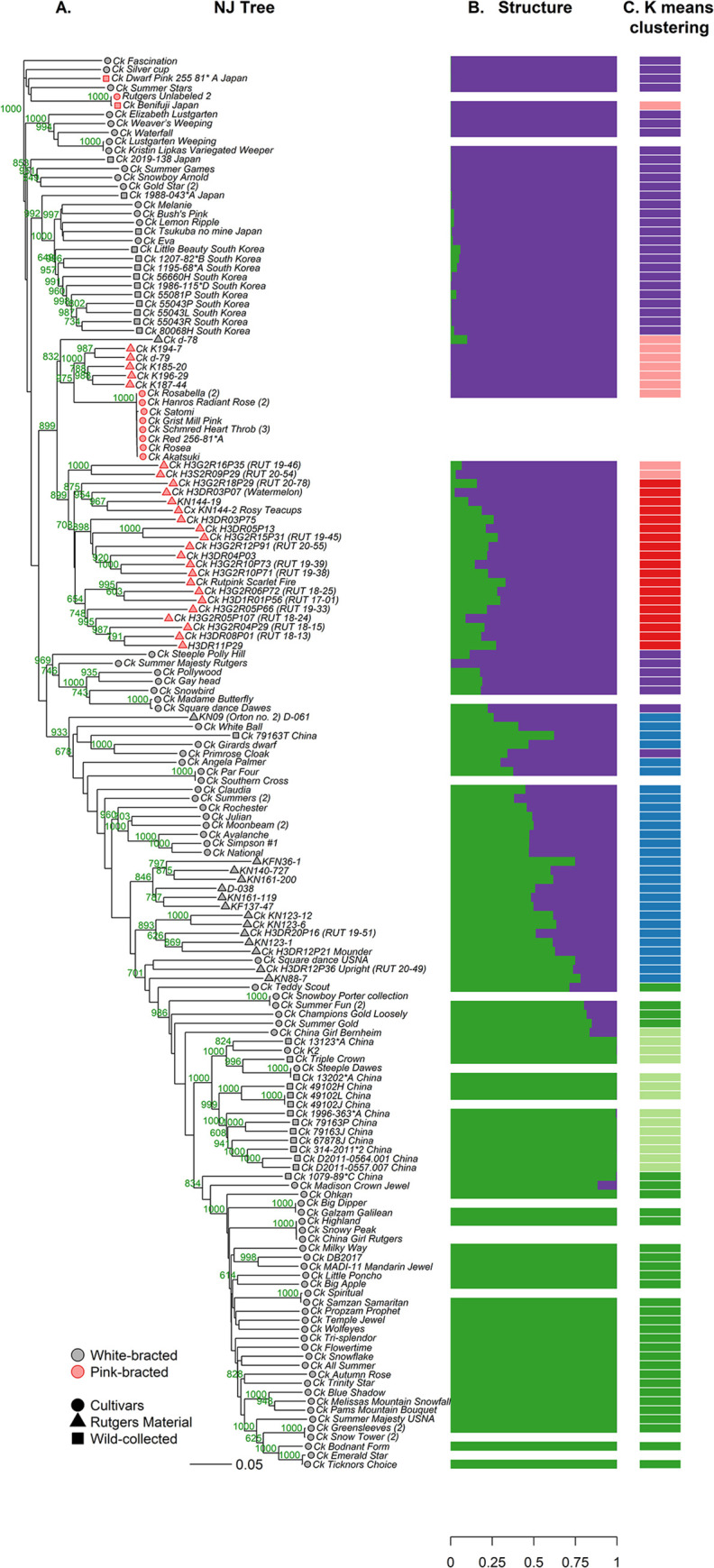
Genetic grouping of 166 *Cornus kousa* dogwood cultivars, breeding selections, and wild-collected accessions with RADseq derived markers. A) Neighbor joining (NJ) tree from the Gower’s dissimilarity matrix of 3,599 single-nucleotide polymorphisms (SNPs) and insertion deletion polymorphisms (Indels). Bootstrap values greater than 600 are indicated in green. Wild-collected accessions’ names are followed by country of origin. Cultivars that were sampled twice from different sources and were clonally identical are followed by (2). Cultivars that that were sampled twice from different sources and were not clonally identical are followed by the name of the source. B) Population structure for genetically unique accessions using mixed-model analysis of top 1000 SNPs and Indels using STRUCTURE software. C) k-means clustering results for genetically unique accessions with 1851 markers (5% or less missing data).

**Fig 6:**
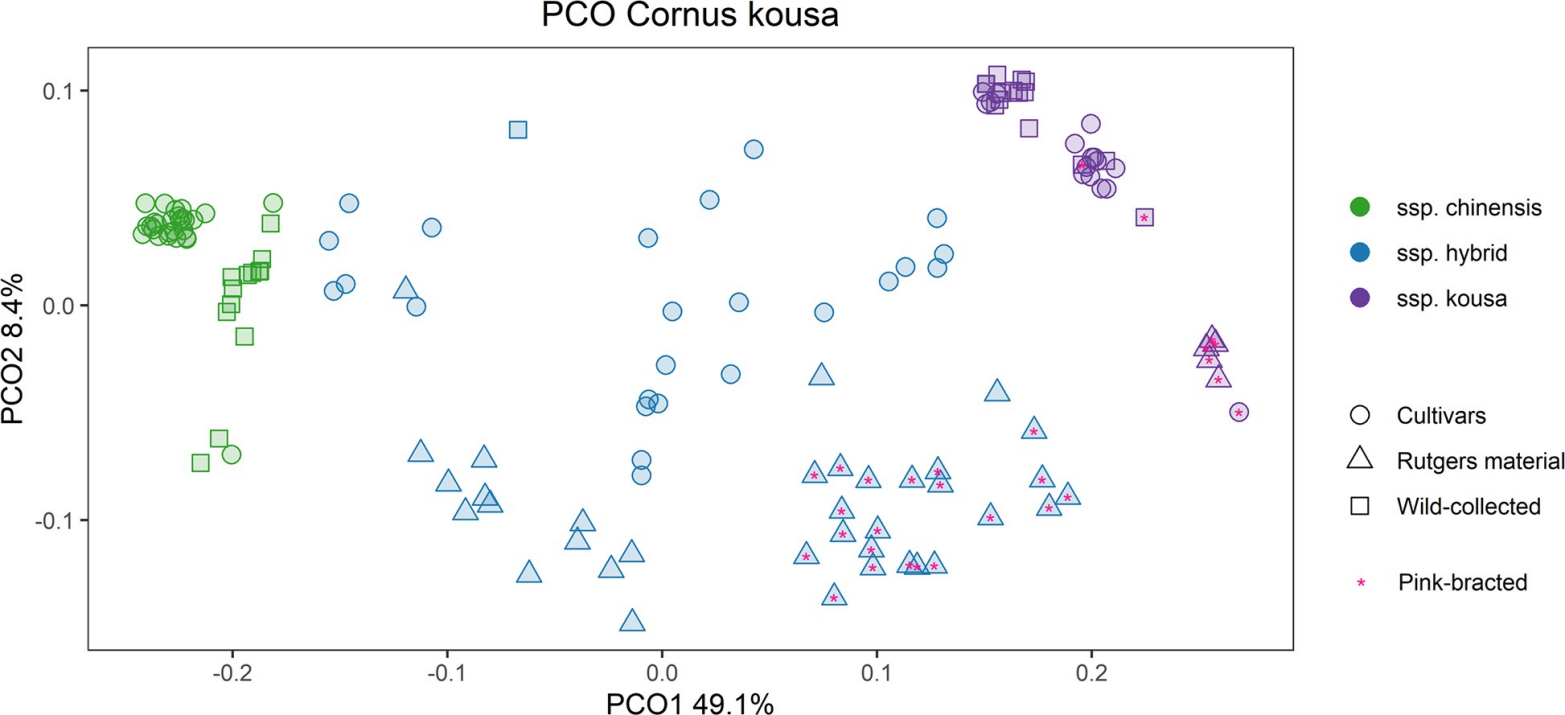
*Cornus kousa* principal coordinate analysis (PCO). The analysis was performed with the same non-duplicate accessions as the STRUCTURE and k-means analyses. Accessions with 90% or greater membership to a ssp. group based on the STRUCTURE results are assigned to that ssp. group in the PCO chart. Accessions with greater than 10% admixture are designated as hybrids.

The subspecies differentiation was supported by the STRUCTURE, NJ tree, PCO, and k-means clustering results (Figs 5 and 6). Although botanically described as subspecies, they are sometimes incorrectly referred to as varieties in the nursery trade. When non-admixed individuals (excluded cultivated hybrids <90% membership to one subspecies) were subjected to AMOVA analysis, 54% of the variation was between subspecies with only 8% variation between individuals within subspecies, which also supports a large genetic differentiation between the two populations (S4 Table). The proportion of variation observed between subspecies in this study (54%) is much higher than the results of a previous SSR genetic diversity and phylogeography study exclusively focused on wild-collected C. kousa (9%) [13]. Both studies determined an optimal STRUCTURE K value of 2, however, in the present study, the assigned subpopulations corresponded more neatly to country of origin and subspecies without admixture being observed within wild-collected accessions. The differences may be due to the increased power of using more, albeit biallelic markers in diversity analysis and population structure differentiation.

Recent RADdseq *Cornus* phylogeny investigations have also shown very clear genetic separation of the ssp. *chinensis* and ssp. *kousa* to the point where the authors considered the evidence for splitting into two separate species, but concluded that subspecies is the correct designation due to possible gene flow, morphological similarities, and relatively recent evolutionary divergence [40, 76]. The current study reveals extensive admixture is present in cultivars suggesting very little reproductive barrier consistent with the subspecies designation. This aligns with empirical observations in the Rutgers dogwood breeding program, as the two subspecies are extremely similar morphologically and intercross readily.

#### *Cornus kousa* ssp. *Chinensis*

The green population in the STRUCTURE results corresponded to ssp. *chinensis* (Fig 5B). The NJ tree (Fig 5A) and PCO analysis (Fig 6) separated the ssp. *chinensis* into two main groups, one consisting primarily of cultivars, the other consisting primarily of wild-collected plants from China. For the cultivars, ‘Galzam’ Galilean®, ‘Milky Way’, ‘China Girl’, ‘Temple Jewel’, ‘Samzan’ Samaritan®, ‘Propzam’ Prophet™, and ‘Greensleeves’ were described as ssp. *chinensis* or likely ssp. *chinensis* by Cappiello and Shadow [1]. In addition, ‘Autumn Rose’ was listed as ssp. *chinensis* by Gayraud [77]. These all fell into the cultivar ssp. *chinensis* group along with 23 other cultivars that were previously unknown to be ssp. *chinensis*: ‘Triple Crown’, ‘Madison Crown Jewel’, ‘Ohkan’, ‘Big Dipper’, ‘Highland’, ‘Snowy Peak’, DB2017, ‘MADI-11’ Mandarin Jewel®, ‘Little Poncho’, ‘Big Apple’, ‘Spiritual’, ‘Wolfeyes’, ‘Tri-Splendor’, ‘Flowertime’, ‘Snowflake’, ‘All Summer’, ‘Trinity Star’, ‘Blue Shadow’, ‘Melissa’s Mountain Snowfall’, ‘Pam’s Mountain Bouquet’, ‘Snow Tower’, ‘Bodnant form’, ‘Emerald Star’, and Ticknor’s Choice’. However, some of the ssp. chinenses cultivars were highly genetically similar (GS> 0.995) suggesting that they are clones. There were clonal groupings of ‘China Girl’, ‘Snowy Peak’, and ‘Highland’; ‘Big Dipper’ and ‘Galzam’ Galilean®; ‘Spiritual’ and ‘Samzan’ Samaritan®; ‘Emerald Star’ and ‘Ticknor’s Choice’; and ‘Greensleeves’ and ‘Snow Tower’.

The boundary between the hybrids and ssp. *chinensis* differed slightly between the STRUCTURE analysis and K-means clustering. The k-means clustering (Fig 5C) also designated ‘Loosely’ Champion’s Gold, ‘Summer Gold’, ‘Summer Fun’, and ‘Teddy Scout’ as ssp. *chinensis* cultivars (dark green population). These four plants are the leftmost labeled ssp. hybrid accessions in the PCO graph (Fig 6) and could be considered ssp. *chinensis* cultivars depending on where the cutoff for ssp. hybrid vs ssp. *chinensis* is determined. The most eastern-collected Chinese accession, 1079–89*C, from Tianmushan Biosphere Reserve in Zhejiang Province was an intermediate accession between the ssp. *chinensis* cultivar group and the ssp. *chinensis* wild-collected group (Fig 5A).

The other ssp. *chinensis* NJ tree group (Fig 5A) and k-means clustering group (light green, Fig 5C) consisted mostly of wild-collected plants from China sampled from ex situ arboreta collections. The Arnold Arboretum’s 13123*A and 13202*A are the oldest accessions in the study, dating back to the early 1900’s. 13123*A is from seed collected by famed plant collector Ernest H. Wilson in 1908 from Yichang in Western Hubei, and 13202*A was received in 1921 from plant collector Joseph Hers. The initially sampled ‘Steeple’ in this group was genetically indistinguishable from wild-collected 13202*A. This similarity likely represents a mix-up of ‘Steeple’ and wild-collected 13202*A in the arboreta collections. The original ‘Steeple’ was selected by gardener Polly Hill. When ‘Steeple’ was resampled directly from the Polly Hill Arboretum it was a hybrid and grouped much closer to the ssp. *kousa* accessions (Fig 5A). The only distinct named cultivar in the mostly wild-collected ssp. *chinensis* group, ‘Triple Crown’, was a Polly Wakefield selection from Arnold Arboretum OP seed [78, 79]. Lastly, an important Rutgers breeding parent, K2, closely grouped with Ernest Wilson’s 13123*A in the NJ tree (Fig 5A). Historical and genetic data suggest a close relationship of the two plants, with K2 as a possible grandprogeny of 13123*A. K2 was acquired as a seedling for Rutgers Horticulture Farm No. 1 (present day Rutgers Gardens) from Willowwood Arboretum (Far Hills, NJ) by Dr. Ben Blackburn in 1949 [7, 80]. Willowwood’s *C*. *kousa* trees were likely sourced from Ernest H. Wilson, who corresponded and exchanged seeds and plants with Willowwoods cofounder, Robert Tubbs, from 1926–1930 [81]. Wilson also invited Tubbs to the Arnold Arboretum in September 1928 to collect seeds. K2 was one of Dr. Orton’s most widely used *C*. *kousa* parents and the female parent for all cultivars of interspecific crosses with *Cornus florida*: ‘Rutlan’ Ruth Ellen®, ‘Rutdan’ Celestial®, ‘Rutcan’ Constellation®, ‘Rutgan’ Stellar Pink®, ‘Rutban’ Aurora®, and ‘KF1-1’ Saturn® [7]. These hybrids are therefore more specifically *C*. *kousa* ssp. *chinensis* × *C*. *florida*.

#### *Cornus kousa* subspecies *hybrids*

Many cultivars showed admixture between the two subspecies. In the STRUCTURE results (Fig 5B), these individuals have less than 90% membership to either the ssp *chinensis* (green) or ssp. *kousa* (purple) population. In the PCO analysis (Fig 6), they also fall intermediate (shown in blue) between the pure ssp. *kousa* and ssp. *chinensis* for the gradient of the first principal coordinate, which explains 49.1% of the variation. In the NJ tree (Fig 5A) most of these admixed accessions form successive outgroups of the ssp. *chinensis* clade and bridge the ssp. *chinensis* and ssp. *kousa*. Only one wild-collected accession, 79163T, showed significant admixture (~40% ssp. *kousa*). The accession notes specify that it is from seed collected in 2008 from Hong De Gu Forest Park in Mei Xian County, Shaanxi province, China. However, this is most likely a mislabeled accession in the arboretum, because when two other trees (79163P and 79163L) from the same seed parent were sampled, they also grouped with the ssp. *chinensis* wild-collected trees (Fig 5A).

#### *Cornus kousa* ssp. *Kousa*

For ssp. *kousa*, wild-collected plants from South Korea grouped in the NJ tree (BS = 649) (Fig 5A), whereas wild-collected plants from Japan were spread out throughout the ssp. *kousa* accessions.

All five weeping accessions grouped together with strong bootstrap support (1000, ‘Elizabeth Lustgarten’, ‘Kristin Lipka’s Variegated Weeper’, ‘Lustgarten Weeping’, ‘Waterfall’, and ‘Weaver’s Weeping’). This suggests that the weeping trait in these cultivars is from a single source and is ssp. *kousa* in origin. ‘Kristin Lipka’s Variegated Weeper’ has been described as a sport of either ‘Lustgarten Weeping’ or ‘Elizabeth Lustgarten’, both weeping forms selected from the same batch OP of seedlings by Jim Cross [1]. In this study, ‘Kristin Lipka’s Variegated Weeper’ was genetically identical to ‘Lustgarten Weeping’, evidence that this was the source of the variegated sport. In contrast to the weeping trait, dwarfing and variegation were two ornamental traits that occur throughout the NJ tree, likely due to their more frequent spontaneous occurrence, or ability to be more easily identified when they appear in a field of nursery stock.

Most of pink-bracted *C*. *kousa* accessions grouped together with high bootstrap support (899). The only accessions that did not fall in this group were the Japanese trees ‘Benifuji’ and ‘Dwarf Pink’. ‘Benifuji’ and ‘Dwarf Pink’ (255–81*A) were selected from wild-collected seed collected from Mount Fuji and Gumma Prefecture, respectively [1, 82]. ‘Benifuji’ has small, dark pink bracts and ‘Dwarf Pink’ has slender pink bracts that vary greatly in color intensity by year, with a dwarf stature when grown on its own roots.

Within the main pink-bracted group, there were eight pink-bracted accessions that exhibited clonal similarity with pairwise GS values ranging from 0.996–0.999. Some of the cultivars have long been suspected to be clones as they are phenotypically indistinguishable from each other and were incompatible when crossed [1, 83]. A previous study found ‘Satomi’ (synonym ‘Miss Satomi’), ‘Rosabella’ and ‘Schmred’ Heartthrob® from several sources were genetically indistinguishable using the DNA amplification fingerprinting (DAF) technique [9]. In this study, ‘Grist Mill Pink’, “Rosea’, ‘Hanros’ Radiant Rose®, ‘Akatsuki’, and ‘Red’ 255–81*A also grouped with the cultivars from the above-mentioned DAF study.

According to genetic and historical data, ‘Satomi’ should be considered the original pink-bracted kousa of this clonal group. ‘Satomi’ was selected by Japanese nurseryman Akiri Shibamichi [1]. Shibamichi sent ‘Satomi’ to Maryland’s Brookside Gardens in 1980 or 1981 before it was officially named ‘Satomi’ (Phil Normandy, Brookside Gardens, personal communication). Brookside Gardens in turn sent ‘Satomi’ to several nurseries and arboreta for evaluation, which is likely when it got introduced to the trade as the rename ‘Rosabella’ (Phil Normandy, Brookside Gardens, personal communication). The modern ‘Rosabella’ and the Morris Arboretum’s ‘Rosabella’ from 1993 (1993–96*B) were genetically identical to the ‘Satomi’ group. Additionally, the Arnold Arboretum’s 256–81*A was one of these Brookside Gardens distributed ‘Satomi’ plants, although it was only listed in the Arboretum’s records as ‘Red’. A photo of 256–81*C (the same clone as 256–81*A) shows inflorescences with bracts very similar to the rest of the ‘Satomi’ clones shown in S4 Fig, although the photo was taken in a different state, year, and time of season, which affects the bract color and size. As additional evidence, Shibamichi selected the variegated ‘Akatsuki’ from a ‘Satomi’ sport, and it is also genetically indistinguishable from ‘Satomi’ as one would expect from a variegated sport. This information verifies the identity of the original ‘Satomi’ in several different ways. It is less clear why the other cultivars are indistinguishable. ‘Schmred’ Heartthrob® (identical when sampled from 3 sources) was patented as being distinct from ‘Satomi’ [84]. ‘Hanros’ Radiant Rose® (identical from 2 sources) is described as having darker pink color and deeper green foliage [1], but both cultivars are phenotypically indistinguishable from others in this clonal group when grown in the Rutgers cultivar trial.

Pink-bracted Rutgers breeding program selections make up the largest subclade within the pink-bracted *C*. *kousa* clade. Dr. Orton used ‘Satomi’, ‘Rosabella’, ‘Rosea’ and ‘Benifuji’ extensively in his breeding program in his quest to develop kousa with darker pink bracts. Because the first three of these accessions are clones, this resulted in frequent backcrossing to the ‘Satomi’ genotype. The large contribution of the Satomi genotype to these breeding selections is likely the reason they grouped in the pink bracted Satomi clade instead of with other ssp. hybrids, even though subspecies status is the strongest genetic delineator for *C*. *kousa* in this study. The most recent accessions with the deepest pink bracts were recognized as a distinct population by admixture (dark pink population in Fig 5C). Within this subclade, closely related trees formed groupings, for example, ‘Rutpink’ Scarlet Fire® with its progeny H3G2R06P72 (RUT 18–25) and H3D1R01P56 (RUT 17–01), as well as ‘KN144-2’ Rosy Teacups® with its OP sibling KN144-19, progeny H3DR03P07, and grandprogeny H3G2R18P29 (RUT 20–78).

Pink bracts have so far only been observed in wild-collected Korean plants [85], wild-collected Japanese plants (‘Benifuji’, ‘Dwarf Pink’), Japanese origin cultivars (‘Satomi’), and in plants derived from the pink Japanese plants (Rutgers breeding selections), making this trait ssp. *kousa* in origin. The Rutgers dogwood breeding program is the oldest continuous *Cornus* breeding program in the world, and the plant material originating from this program has begun to develop a unique genetic signal. In the PCO analysis (Fig 6), the second principal coordinate (8.4%) separated out Rutgers breeding material from most other wild-collected and cultivated material. Additionally, ssp. *kousa* ‘Satomi’ and ssp. *chinensis* K2 had similar negative PC2 values as the Rutgers breeding material (Fig 6), validating their outsized importance as parents in the Rutgers dogwood breeding program. Nearly all the Rutgers breeding selections were subspecies hybrids with the white-bracted selections having a greater share of ssp. *chinensis* than the pink-bracted.

Several other dogwood breeders have made their mark on the kousa germplasm. Horticulturalist Polly Hill’s selections ‘Steeple’ (sampled from the Polly Hill Arboretum), ‘Pollywood’, ‘Gay Head’, ‘Snowbird’ and ‘Square Dance’ formed a clade (BS = 969) and were all subspecies hybrids with 76–84% ssp. *kousa*. ‘Square Dance’ sampled twice from the USNA and Dawes Arboretum were distinct, however, the Dawes arboretum clone is likely the correct one because it was directly sourced from the Polly Hill Arboretum and was placed in the Polly Hill clade. Hill also selected a ssp. hybrid ‘Julian’ which genetically presents like an F_1_ hybrid (most distinguishing ssp. *kousa* vs ssp. *chinensis* loci are heterozygous) and two pure ssp. *chinensis* cultivars, ‘Big Apple’ and ‘Blue Shadow’. Hill’s legacy continues with seed she gifted to the University of Tennessee. Their recent releases ‘Pam’s Mountain Bouquet’ and ‘Melissa’s Mountain Snowfall’ were selected from the gifted seed lot and formed a clade with ‘Blue Shadow’, indicating that it is the likely seed source. Dogwood enthusiast Mary (Polly) Wakefield’s selections ‘Greensleeves’, ‘Moonbeam’, ‘Silver Cup’, and ‘Triple Crown’ are much more genetically disparate from each other, presenting as ssp. *chinensis*, ssp. hybrid, ssp. *kousa*, and ssp. *chinensis*, respectively. Her selection ‘Greensleeves’ is considered one of the best white-bracted *C*. *kousa* cultivars and still widely sold today.

#### Parentage analysis

Triad analysis with apparent confirmed that three of Rutgers’ pink-bracted breeding selections, K187-44, Ck_K196-29, Ck_K194-7, were the progeny of ‘Satomi’(‘Rosabella’) × ‘Benifuji’ (p = 0.012–0.035, GD = 0.007–0.009). Another pedigreed progeny of ‘Benifuji’ and ‘Satomi’, K185-20, was also below the gap threshold at GD = 0.011 but was not designated as a significant outlier by the second Dixon test. Also with GD = 0.011 was a trio involving ‘Benifuji’, ‘Satomi’, and D-79, an additional pink-bracted breeding selection with an uncertain pedigree, which also wasn’t significant in the second Dixon test, however, these two accessions are likely both progeny of ‘Satomi’ and ‘Benifuji’ because of their placement with the significant accessions in the NJ tree (Fig 5A), next to ‘Satomi’.

Triad analysis assigned ‘Triple Crown’ as the progeny of Arnold Arboretum’s Chinese collected 13123*A and 13202*A (p<0.0001, GD = 0.004), explaining its position next to the accessions in the wild-collected ssp. *chinensis* group. In addition, the accession DB2017 was the progeny of ‘MADI-II’ Mandarin Jewel® and ‘Blue Shadow’ (p = 0.018, GD = 0.008).

The gap between true and spurious triads was much smaller in the *C*. *kousa* analysis (S5A Fig) than in the *C*. *florida* analysis (GD gap between 0.011–0.016 vs 0.003–0.023, respectively). This was driven by two factors. The full sibling progeny of ‘Benifuji’ × ‘Satomi’ were designated as possible parents in the analysis to test if they were also OP parents of other Rutgers breeding accessions, however, different triad combinations of the full siblings as parents of each other in combination with ‘Benifuji’ or ‘Satomi’ had GD values ranging from 0.016–0.024. In this range there were a few triads involving ssp. *chinensis* cultivars. The strong subspecies delineation may have artificially decreased the GD values of potential triads that belonged to the subspecies *chinensis* cultivar group, as there weren’t many distinguishing homozygous loci between them.

The dyad analysis did not return a significant gap between *P*_*OHL*_ 0.01–0.02 (S5B Fig). Upon closer inspection of the data, many pedigreed relationships of Rutgers advanced backcross hybrids (0.5–7% *C*. *nuttallii* in *C*. *kousa* backgrounds) fell in this zone, which is higher than one normally would accept to validate parent progeny relationships. This may signify a slightly higher genotyping error for the advanced interspecific hybrids e.g. heterozygote loci being called as homozygous loci, which would increase the *P*_*OHL*_ values when comparing the putative parent progeny pairs. Despite this, the advanced backcross hybrids generally grouped with their progeny and other close relatives (ex. full siblings, grandprogeny) in the NJ tree. Additionally, as seen in the triad analysis, the strong subspecies delineation may have artificially decreased the *P*_*OHL*_ values of potential parent progeny pairs that belonged to the same subspecies group, as there were many unverifiable pairs that seeped down into the 0.01–0.02 *P*_*OHL*_ region. Therefore no dyads were considered significant according to the dyad analysis. However, possible true dyads with *P*_*OHL*_ > 0.010 are listed in S5 Table and their parentage could be further investigated.

The apparent’s dyad analysis was originally considered for use because it promised accurate parentage assignment even when other closely related individuals were included in the dataset as possible parents [41]. It approaches the analysis through a two-stage statistical test to determine if an accession is more closely related to a population of its hypothetical siblings than to a population of random individuals, then tests if the variation in GD is higher between the accession and a population of its siblings than between an accession and a population of its siblings’ progeny. However in practice, for both species, apparent’s dyad analysis returned as many “significant” dyads with 0.02 < *P*_*OHL*_ < 0.10 as *P*_*OHL*_ < 0.01, in addition to missing dyads that could be significant with *P*_*OHL*_ < 0.01. This underlies the necessity for additional development of dyad parentage analysis tools for SNP and Indel markers or perhaps specific biallelic marker filtering strategies. If parentage analysis is the main goal in a study, then highly polymorphic SSR markers may be a better choice for evaluating parentage of highly related individuals, especially when the markers were filtered over a dataset with several subspecies or populations.

#### KASP genotyping panel

**Fig 7:**
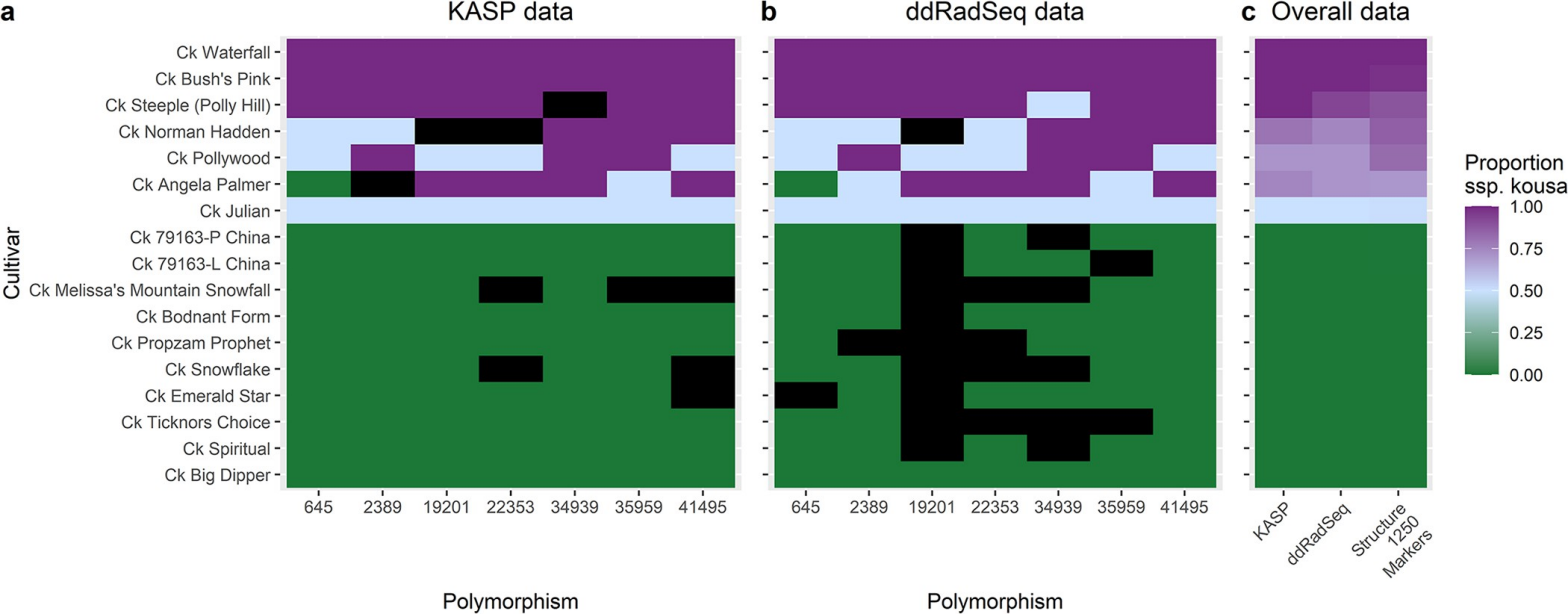
KASP genotyping panel results for subspecies determination of *Cornus kousa* accessions. a) KASP data for seven marker assays. b) RADseq data for the same seven markers. For a) and b), green indicates homozygotes for the ssp. *chinensis* allele (value of 0), purple indicates homozygotes for the ssp. *kousa* allele (value of 1), light blue indicates heterozygotes (value of 0.5), and black is missing data. c) Heat map of overall data comparing the averages of marker values for the 7 KASP assays and 7 RADseq markers with STRUCTURE results using top 1000 markers.

A KASP genotyping panel with seven KASP assays was developed for distinguishing ssp. *chinensis*, ssp. *kousa*, and ssp. hybrids as the groups are difficult to distinguish phenotypically. The KASP genotyping panel was 100% in agreement with the RADseq genotyping calls for the corresponding markers excluding missing data (Fig 7). This is in line with high accuracy estimates (>98.4%) for KASP [86]. Additionally, the seven-marker KASP genotyping panel accurately determined the subspecies assignment for 15 out of 16 unknown trees (94%) when compared to STRUCTURE analysis with 1000 markers (Fig 7). The only exception was the designation of the unknown ‘Steeple’ (Polly Hill Arboretum) as 100% ssp. *kousa*, while the STRUCTURE results designated it as a hybrid with 89% ssp. *kousa*.

Developing a panel with multiple assays was necessary for accurate subspecies assignment; hybrids can be complex mixes of ssp. *kousa* and ssp. *chinensis* with different combinations of species differentiating loci being homozygous for ssp. *kousa* alleles, homozygous for ssp. *chinensis* alleles, or heterozygous. Using all seven assays in the genotyping panel is recommended for the most accurate results. Results should be interpreted as the following: 0–10% ssp. *kousa* alleles = ssp. *chinensis*, 10–90% ssp. *kousa* alleles = ssp. hybrid, and 90–100% ssp. *kousa* alleles = ssp. *kousa*. Between 10–90%, the value can be viewed as a rough estimate for the percentage of ssp. *kousa* in the sample.

This panel will be useful to arboreta and breeders for determining subspecies assignment of historic *C*. *kousa* cultivars that were unable to be included in this study, as well as future cultivars with limited pedigree information. The advantage of the genotyping panel over RADseq for subspecies assignment of a few samples is speed, cost-effectiveness and simplicity of data processing and analysis.

### Advanced interspecific hybrids in the Rutgers University breeding program

Famed Rutgers dogwood breeder Dr. Elwin Orton pioneered hybridization of *C*. *kousa × C*. *florida* (*C*. × *rutgersensis*) and *C*. *kousa* × *C*. *nuttallii* (*C*. *× elwinortonii*) [7]. Over his career he released multiple cultivars marketed under the Stellar Series® name, most of which were sterile F_1_ hybrids of *C*. *kousa × C*. *florida;* he also released one semi-fertile hybrid reported to be a backcross to *C*. *kousa* (*C*. × *rutgersensis ’*KF111-1’Hyperion® dogwood). Further, he released the F_1_ *C*. *kousa* × *C*. *nuttallii* ‘KN4-43’ Starlight® dogwood and the reported backcross to *C*. *kousa*, ’KN30-8’ Venus® dogwood. In continuation of breeding efforts, Dr. Orton backcrossed the rare semi-fertile F_1_ hybrids to *C*. *kousa* over several generations and intercrossed select plants of mixed interspecific origin sometimes including all three species in their pedigrees. He performed controlled crosses in addition to collecting OP seeds. Through generations, he tracked hybrid status with combinations of the letters K, F, and N in tree identifier codes (for Kousa, Florida, and Nuttallii, respectively). According to Rutgers University pedigree records, about half of the current Rutgers breeding selections, although phenotypically *C*. *kousa*, likely hold small amounts of introgression of *C*. *nuttallii* and/or *C*. *florida*. Moreover, due to the growing of OP seed of select interspecific hybrid trees inter-pollinating in the breeding blocks (sometimes over multiple generations), there is likely additional mixing of genetic backgrounds. However, most of the modern breeding accessions are phenotypically indistinguishable from pure *C*. *kousa* accessions (fruit type, bloom phenology, leaf size and shape, floral bract shape) except for darker pink bract color. One hypothesis considered in our breeding program to explain the darker color was the possible presence of *C*. *florida* genes, as *C*. *florida* bracts can achieve a deep red color and many of the dark pink breeding selections have *C*. *florida* in their pedigrees.

**Fig 8:**
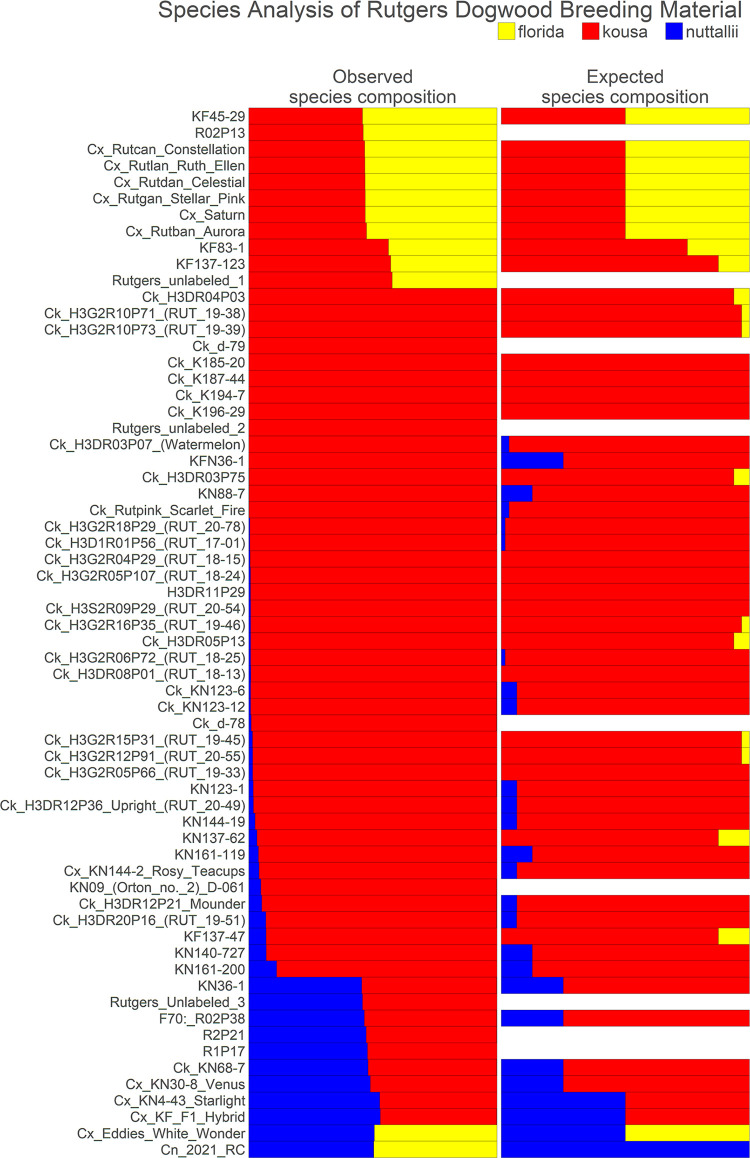
Expected vs observed species composition of 74 Hybrids and Rutgers Dogwood breeding selections. The right panel shows expected species makeup based on pedigree records and left panel shows the calculated species results. Blank spaces in the expected panel denote accessions with unknown pedigrees.

To determine the interspecific hybrid composition of advanced selections from the Rutgers breeding program, the analysis was rerun from the GBS-SNP-CROP pipeline step. Species-specific SNPs and Indels determined from reference samples of each species—10 *C*. *florida*, 10 *C*. *kousa*, and 6 *C*. *nuttallii*—were used to analyze hybrid makeup of known and suspected hybrids and Rutgers *C*. *kousa* breeding selections and compared to expected hybrid makeup based on pedigree (Fig 8).

**Fig 9:**
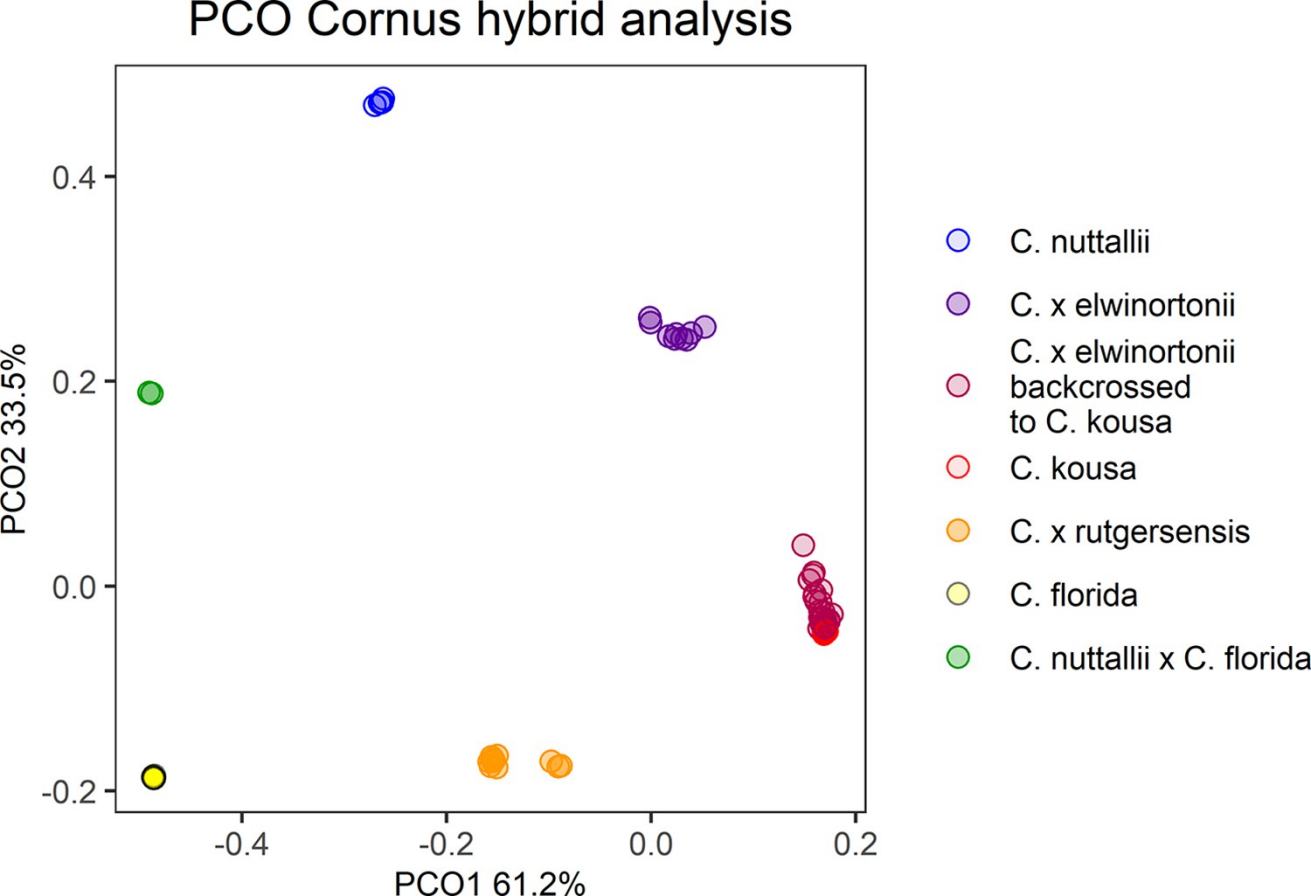
PCO based on the Gower’s dissimilarity matrix of 8109 markers for determining the species composition of hybrids and breeding material. The 74 hybrids and *C*. *kousa* Rutgers breeding selections are shown with the 26 reference samples.

**Fig 10:**
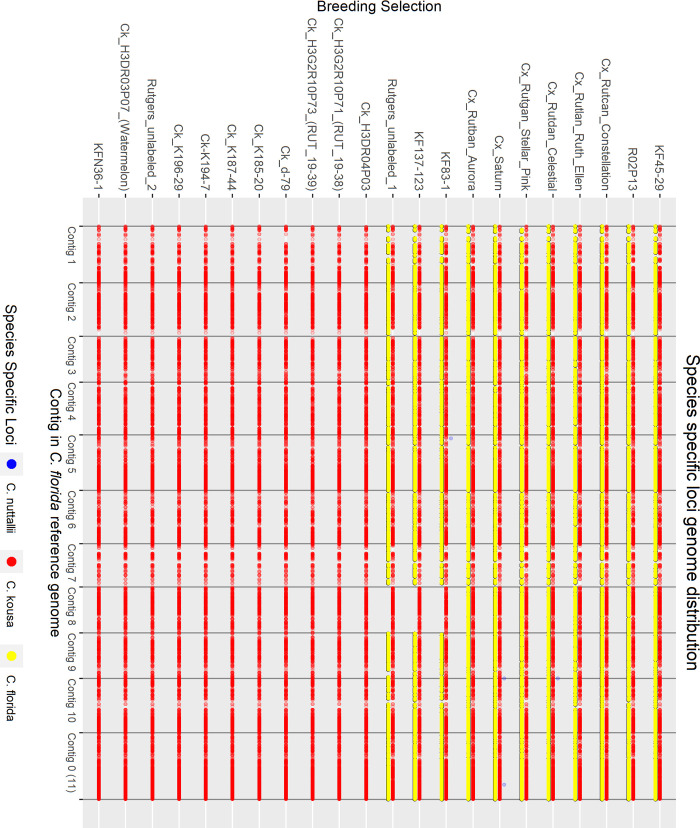
8109 species specific loci genome distribution for *Cornus* hybrids and Rutgers breeding lines 1. The first third of accessions from Fig 8 are shown. Markers are arranged according to their synteny with the *Cornus florida* ‘Appalachian Spring’ reference genome.

**Fig 11:**
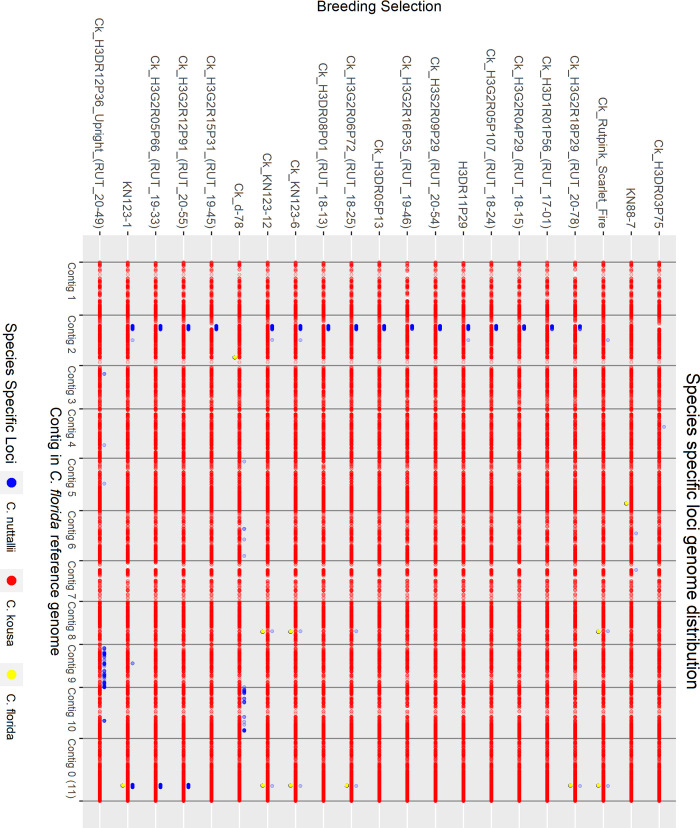
8109 species specific loci genome distribution for *Cornus* hybrids and Rutgers breeding lines 2. The second third of accessions from Fig 8 are shown. Markers are arranged according to their synteny with the *Cornus florida* ‘Appalachian Spring’ reference genome.

**Fig 12:**
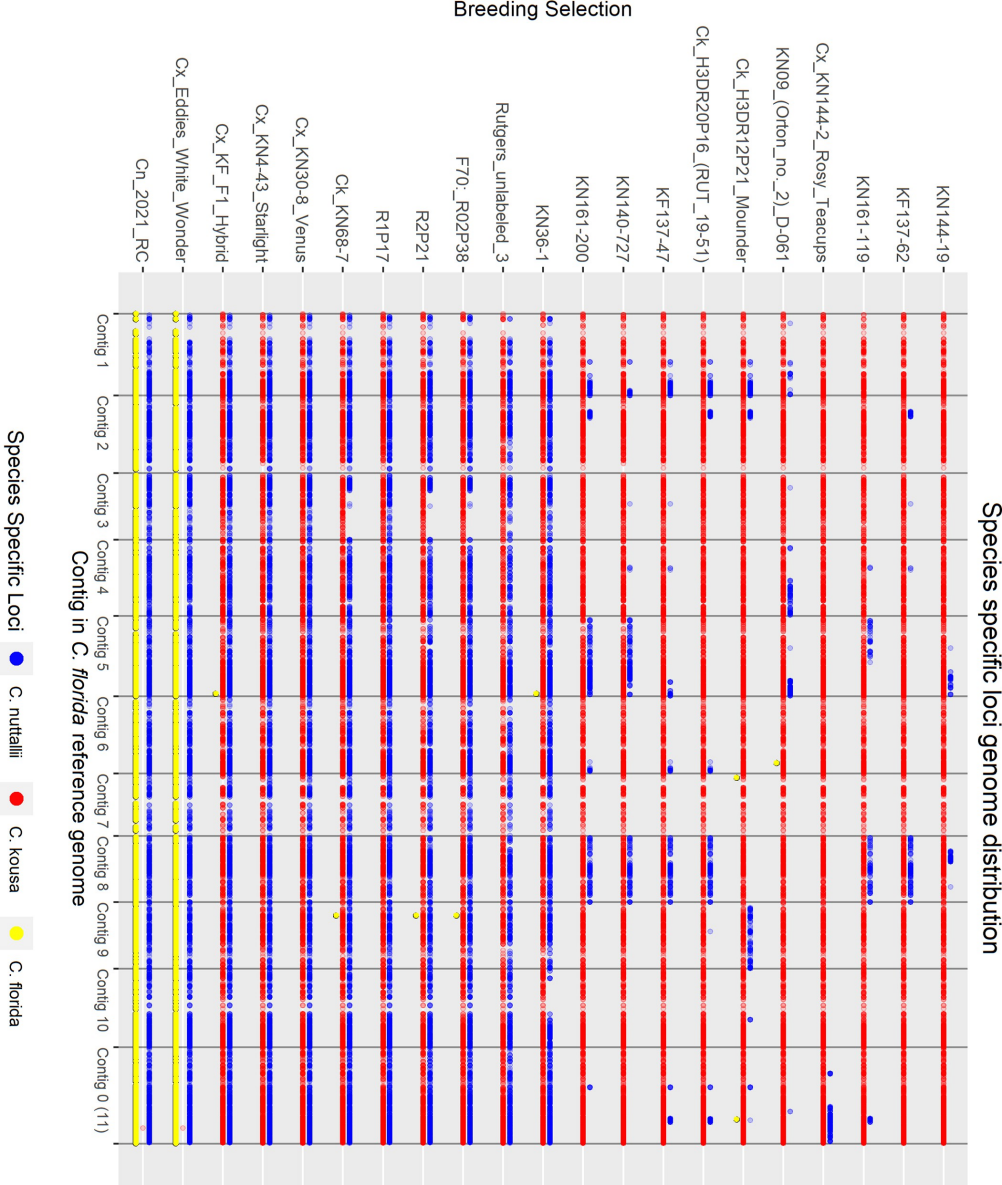
8109 species specific loci genome distribution for *Cornus* hybrids and Rutgers breeding lines 3. The last third of accessions from Fig 8 are shown. Markers are arranged according to their synteny with the *Cornus florida* ‘Appalachian Spring’ reference genome.

First generation hybrid status was validated for the Rutgers *C*. *× rutgersensis* (*C*. *kousa* × *C*. *florida*) hybrids ‘Rutgan’ Stellar Pink®, ‘Rutcan’ Constellation®, ‘Rutdan’ Celestial®, ‘Rutban’ Aurora®, ‘Rutlan’ Ruth Ellen®, ‘KF1-1’ Saturn® and breeding selection KF45-29, as well as *C*. *× elwinortonii* (*C*. *kousa* × *C*. *nuttallii*) ‘KN4-43’ Starlight®. Additionally, the analysis confirmed that one of the accessions received for the study as *C*. *nuttallii* was in fact a *C*. *florida* × *C*. *nuttallii* hybrid along with the known hybrid ‘Eddie’s White Wonder’. All F_1_ hybrids had close to 50% of each parental species based on the observed species-specific marker frequency (Fig 8), were positioned halfway between the parents in the PCO analysis (Fig 9) and were heterozygous for the species-specific markers along the lengths of their genomes (Figs 10–12).

Some F_1_ hybrid accessions had one or two instances of an unexpected heterozygous species-specific marker (i.e. Celestial® with 1 heterozygous *C*. *nuttallii* marker on Contig 9 and ‘Eddie’s White Wonder’ with 1 heterozygous *C*. *kousa* marker on Contig 0.) These are likely sequencing errors or perhaps loci that are not truly species-specific even though they passed the 26 reference accession filter.

Accessions that were one generation removed from the F_1_ hybrids tended to have relatively equal percentages of both species, instead of the expected 75% backcrossed species and 25% non-backcrossed species. In the case of *C*. × *elwinortonii* ‘KN30-8’ Venus® ([*C*. *kousa* ‘Chinensis’ × *C*. *nuttallii* ‘Goldspot’] × *C*. *kousa* ’Rosea’), this discrepancy is likely due to its triploid nature [87]. The genotyping was unable to discern allele copy number within polymorphic loci to show allele dosage, so the loci were scored as diploid heterozygous instead of triploid heterozygous. Venus® is the only documented triploid big-bracted dogwood, therefore, polyploidy should not be a confounding factor for the rest of the analysis. A closer look at the other hybrid first-generation backcrosses to *C*. *kousa* (ie KN68-7, KF83-1, and KN36-1) showed mostly heterozygous loci along the length of their genomes except for segments of single chromosomes homozygous for *C*. *kousa* alleles (Figs 10 and 12). The heterozygous regions were not the same between all accessions. Even Orton’s most fertile F_1_ hybrids had greatly reduced seed set, germination rate, and subsequent seedling survival compared to the straight species. Perhaps only certain hybrid combinations of chromosomes were able to produce viable progeny that were vigorous enough to be kept in the breeding program and used as breeding parents.

Moving forward through generations in the germplasm, advanced backcross hybrid status was confirmed for many of Orton’s KN accessions. Interestingly, the contribution of the donor parent species tended to be smaller than was expected based on a theoretical halving of the quantity of donor species DNA with each backcross (BC) generation (Figs 11 and 12). For example, KN30-1 (unsampled full sibling of ‘KN30-8’ Venus®) was the parent of OP progeny KN140-727, KN161-119, and KN161-200, which were majority *C*. *kousa* with 7.0%, 3.9%, and 11.2% *C*. *nuttallii*, respectively. The percentage of *C*. *nuttallii* in the three trees was less than but still reasonably close to the theoretical second-generation backcross (BC_2_) values of 12.5%. There was a range in the percentage of *C*. *nuttallii* demonstrating how the amount of donor DNA present in each backcross generation may follow a bell-shaped curve because of random segregation of chromosomes in meiosis. For the next backcross generation (BC_3_), the theoretical percentage of donor DNA is 6.25%. One of KN30-1’s other progeny, KN161-1 (unsampled) was the seed source of OP progeny Ck H3DR12P21 (Mounder), Ck H3DR12P36 Upright (RUT 20–49), and Ck H3DR20P16 (RUT 19–51). These trees were 5.2%, 1.8%, and 6.9% *C*. *nuttallii*, respectively, in predominantly *C*. *kousa* backgrounds. KN30-1’s OP progeny KN71-1 (unsampled) was the parent of OP KN123-1, KN123-6, and KN123-12, which were 1.8%, 0.7%, and 0.7% *C*. *nuttallii*. Additionally ‘KN144-2’ Rosy Teacups®, a BC_3_ from *C*. *× elwinortonii* with controlled crosses to *C*. *kousa* for three generations [88], was 4.1% *C*. *nuttallii* and 95.9% *C*. *kousa*. All these trees had detectible amounts of *C*. *nuttallii* but six out of seven were less than 6.25%. The BC calculations for OP events were made with the assumption that they were pollinations with pure *C*. *kousa*, although many of the plants interpollinating in the collections and breeding fields were also interspecific hybrids of mixed origin. However, if interspecific hybrids were contributing pollen to these crosses, one would expect even more hybrid DNA in these accessions than was observed.

Most recent selections in the Rutgers breeding program (designated (RUT ##-##)) were genetically majority *C*. *kousa* with small introgressions of *C*. *nuttallii* DNA (0.5–7%), concentrated in specific chromosomal regions that were usually shared by at least 3 or more of the tested accessions (Figs 11 and 12). The most common of these regions (14/20 recent breeding selections) was on Contig 2 and spanned at least 9.9 Mb. All individuals with this introgression were heterozygous except for H3G2R15P31 (RUT 19–45), which was homozygous for the *C*. *nuttallii* alleles.

There was not a clear pattern of certain introgression regions being present in recent breeding selections with dark pink bracts. For example, ‘Rutpink’ Scarlet Fire® did not have the contig 2 region shared by most of the recent breeding selections; it was overwhelmingly *C*. *kousa* with only 3 heterozygous *C*. *nuttallii* and 2 *C*. *florida* alleles. There were several other accessions with a few *C*. *florida*-specific alleles, however, these alleles always appeared singly and not in a series with neighboring *C*. *florida*-specific loci. It is therefore difficult to discern if these are truly *C*. *florida* specific alleles, although it cannot be ruled out because many of these plants have *C*. *florida* in their pedigrees or are the result of OP events in fields with advanced backcross hybrids of interspecific origin.

It is possible that some small introgression regions of *C*. *nuttallii* or *C*. *florida* could have been missed because of gaps in genomic coverage with RADseq. Assuming that the reference genome order is correct, for *C*. *florida* specific markers, there were 26 gaps of 5–15.2 Mb and 62 gaps of 3–5 Mb. For *C*. *nuttallii* specific markers, there were 37 gaps of 5–19.4 Mb and 58 gaps of 3–5 Mb. These types of gaps are not unexpected given that RADseq reduces the complexity, and therefore coverage, of the genome by design. These gaps could encompass repetitive regions with few genes or regions that were too divergent between species, for example lacking common restriction enzyme sites, to yield common loci across species.

*Cornus nuttallii* does not have documented variation in bract color from white, however, it blooms a few weeks earlier than *C*. *kousa*. Bract color intensity in *C*. *kousa* is environmentally influenced as well as genetically influenced, with pink-bracted trees showing the best color in springs with cool, wet weather during bract expansion [1]. It is feasible that *C*. *nuttallii* introgressions are being selected for in the Rutgers breeding program with the recent push for slightly earlier-flowering *C*. *kousa* types, whose bracts reach full size and color before the hottest days of spring. Additionally, *C*. *nuttallii* may be contributing to attractive leaf traits such as darker green color and less curling, another Rutgers breeding program goal.

In conclusion, F_1_ hybrids from all three species were confirmed as well as several generations of *C*. *× elwinortonii* backcrosses to *C*. *kousa*. Most recent selections in the Rutgers breeding program were genetically majority *C*. *kousa* with small introgressions of *C*. *nuttallii*. *Cornus nuttallii* introgressions being more common than *C*. *florida* in the breeding material was unexpected given the breeding program’s history of deliberate breeding crosses and open pollination in fields with both *C*. × *rutgersensis* and *C*. *× elwinortonii* interspecific hybrid selections. It is not possible to rule out *C*. *florida* DNA being present in the recent breeding selections and contributing to their unusual dark pink bract color due to a few genomic gaps in the data. However, it is more likely from the available data that the dark pink color is the result of stacking pink bract color alleles and modifying loci from *C*. *kousa*, with additional modifying loci from *C*. *nuttallii*.

## Conclusion

The RADseq-derived SNP and Indel markers were successful for assessing genetic diversity, population structure, subspecies makeup, and parent-progeny relationships for species’ cultivated and ex situ wild-collected germplasm. They were also useful for determining species assignment of hybrids and advanced generation backcrosses when roughly equally sized samples of non-hybrid individuals were used as references. However, bi-allelic markers that passed missing data filtering thresholds when including multiple species in the dataset tended to only differentiate between the species and were uninformative for diversity analysis within species. Therefore, separate variant filtering and marker calling was required with each set of accessions and goals.

When all samples were analyzed together, *C*. *florida* and *C*. *kousa* accessions were clearly differentiated. The PCO analysis distinguished the two species from each other and *C*. *nuttallii*, with hybrids falling intermediate.

For the *C*. *florida* independent analyses, population structure differentiated white bracted from pink- and blush-pink bracted plants (forma *rubra*). The forma *rubra* plants appear to share population structure that may come from a genetic bottleneck as many of the accessions share ‘Cherokee Chief’ as a direct parent. The analysis also differentiated *C*. *florida* ssp. *urbiniana* from ssp. *florida*, even though few ssp. *urbiniana* accessions were included.

For the *C*. *kousa* independent analyses, the results supported *Cornus kousa* ssp. *chinensis* as a genetically distinct subspecies with clear separation from *C*. *kousa* ssp. *kousa* from Japan and Korea. In addition, this study was able to differentiate cultivars into ssp. *chinensis*, ssp. *kousa*, and admixed groups. Our analysis verified seven previously described ssp. *chinensis* cultivars and found an additional eleven nonredundant ssp. *chinensis* cultivars. These cultivars could be evaluated to determine if ssp. *chinensis* is truly a superior ornamental form of the species and if so, could be marketed in the industry as superior. In addition, a KASP genotyping panel with seven assays was developed for fast and easy subspecies assignment with 94% success rate compared to a larger 1000 marker set.

There are many cases of mistaken identity in the trade, especially for desirable pink-bracted cultivars of both species. Many of these cases likely originated with genetically distinct but phenotypically similar cultivars, underlying the need for careful propagation, labeling, and record-keeping in nurseries and plant collections. Additionally, thorough phenotypic and genotypic evaluation of potential cultivars is essential to ensure that only genetically unique and phenotypically distinct cultivars are being introduced. However, this type of rigorous evaluation is lagging for ornamental plants compared to other crops because of a lack of resources.

Despite the significant cultivar synonymy, genetic diversity levels of unique cultivars and wild-collected plants is high, especially for white-bracted trees.

The species-specific marker analysis verified interspecific F_1_ hybrids between *C*. *florida*, *C*. *kousa*, and *C*. *nuttallii* as well as second and third generation backcrosses of C. × *elwinortonii* (*C*. *kousa* × *C*. *florida*) to *C*. *kousa*. Most of the recent selections in the Rutgers breeding program are predominantly *C*. *kousa* with small (0.5–7%) introgressions of *C*. *nuttallii* DNA. Based on the current data, it is more likely that the dark pink color of the recent Rutgers breeding selections is the result of stacking pink bract color alleles and modifying loci from *C*. *kousa*, with additional modifying loci from *C*. *nuttallii* rather than from the contribution of *C*. *florida* alleles.

The results of this diversity study are useful to plant breeders, arboreta, and the industry, as most modern cultivars, popular historic cultivars, and ex situ wild-collected germplasm are represented.

## Supporting information

S1 Fig Neighbor Joining tree of all accessions in the study using 3009 markers. (TIF)

S2 Fig Pink-bracted clade with high bootstrap values when *C*. *florida* ssp. *urbiniana* SMN is excluded from the analysis. (TIF)

S3 Fig *Cornus florida* parentage analyses plots for 7622 markers. a) Triad analysis plot for test triads, ordered by Gower Dissimilarity. b) Dyad analysis plot for test dyads, ordered by POHL. Red dotted lines indicate the halfway point of each significant gap and red data points are the triads/dyads that were tested for significance with a second Dixon test.
(TIF)

S4 Fig Photos of selected pink-bracted *Cornus kousa* cultivars included in the study. The location and date of photos is indicated to the left of each panel. The plants in the gray rectangles clustered together in the NJ tree made from 7,622 SNP and Indel markers and shared between 0.996 and 0.999 similarity based on a modified Gower’s similarity metric. Photos are not to scale.
(TIF)

S5 Fig *Cornus kousa* parentage analyses plots for 3599 markers. a) Triad analysis plot for test triads, ordered by Gower Dissimilarity. b) Dyad analysis plot for test dyads, ordered by POHL. Red dotted lines indicate the halfway point of each significant gap and red data points are the triads/dyads that were tested for significance with a second Dixon test.
(TIF)

S1 Table List of arboreta, nurseries, and private collections where *Cornus* trees were sampled for this study. (XLSX)

S2 Table Study accession list with background information. (XLSX)

S3 Table *Cornus kousa* subspecies KASP primers and summary information. (XLSX)

S4 Table Analysis of molecular variance (AMOVA) of *Cornus kousa* ssp. *kousa* and *Cornus kousa* ssp. *chinensis*. (XLSX)

S5 Table Possible *C*. *kousa* dyads based on P_OHL_ > 0.010 for 3599 markers. Dyads from triads already deemed significant were excluded.
(XLSX)

S1 Text Supplementary discussion. Additional information about synonymous cultivars of *C*. *florida* and *C*. *kousa*, *C*. *florida* parent-progeny relationships, and hybrid analysis sibling groups.
(DOCX)
