# Supplementary figures and images for: Genetic diversity analysis of big-bracted dogwood (Cornus florida and C. kousa) cultivars, interspecific hybrids, and wild-collected accessions using RADseq
Source: PLoS One. 2024 Jul 25;19(7):e0307326. doi: 10.1371/journal.pone.0307326 (PMC11271954; doi:10.1371/journal.pone.0307326)

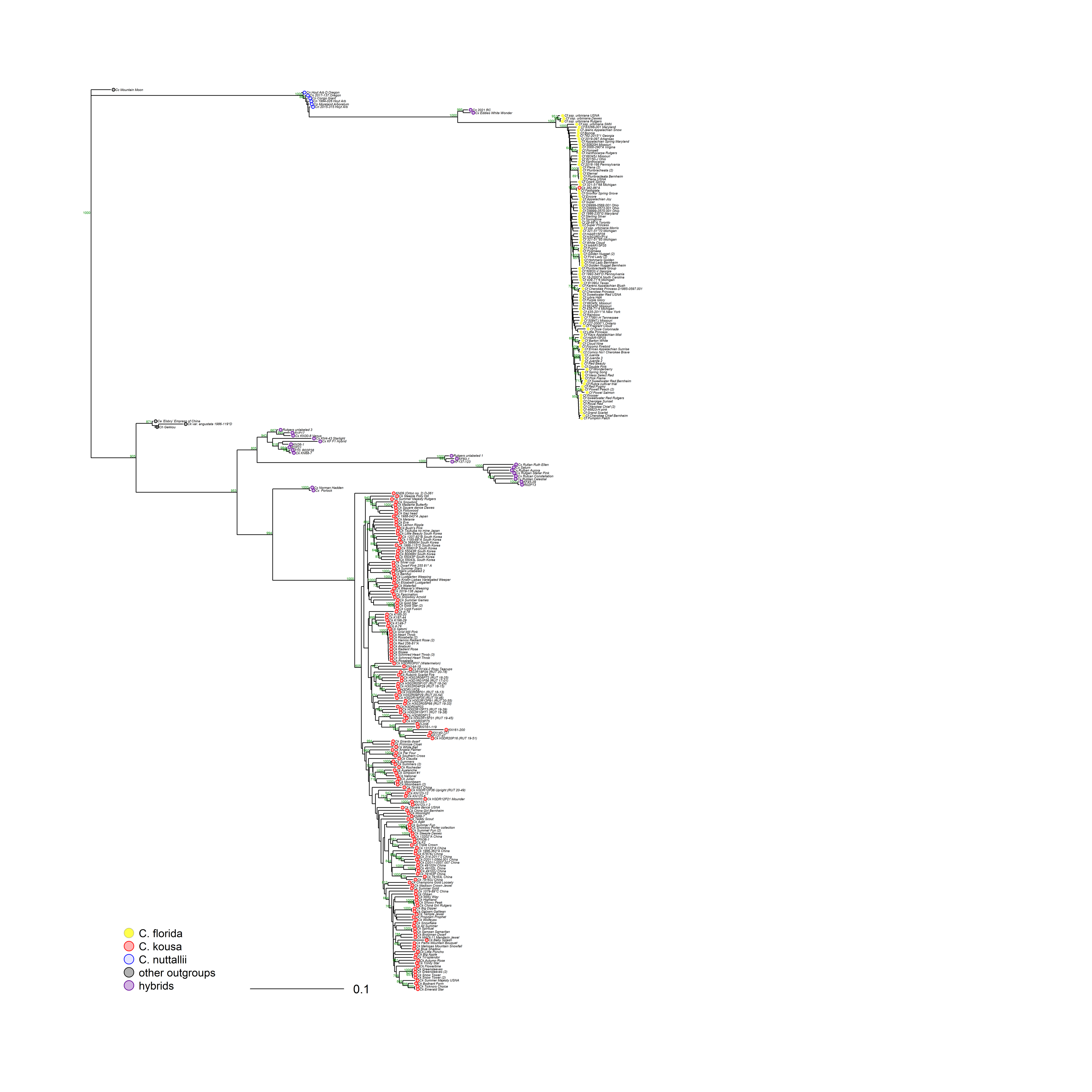

Supplement: S1 Fig — (TIF) [file pone.0307326.s001.tif]

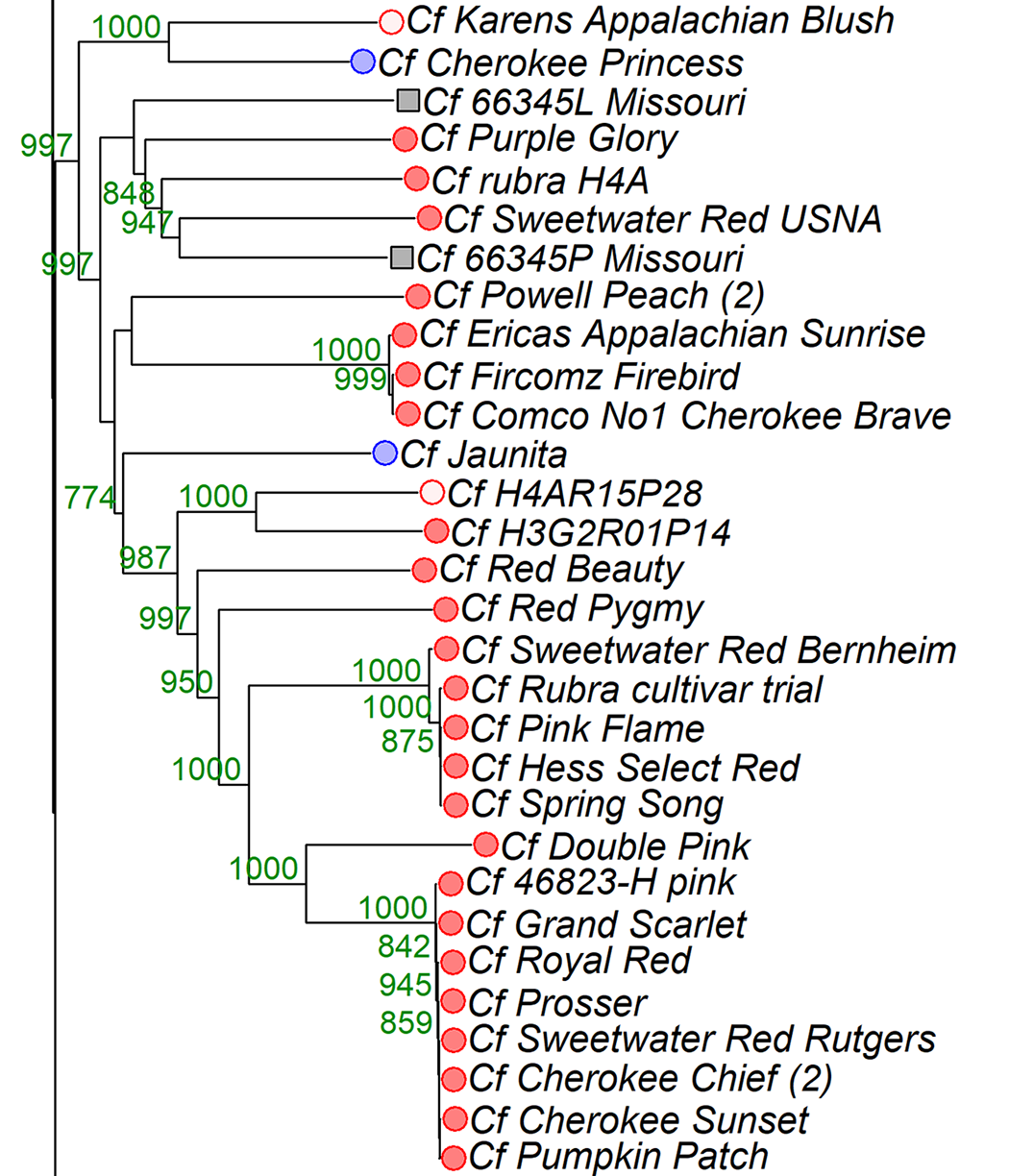

Supplement: S2 Fig — (TIF) [file pone.0307326.s002.tif]

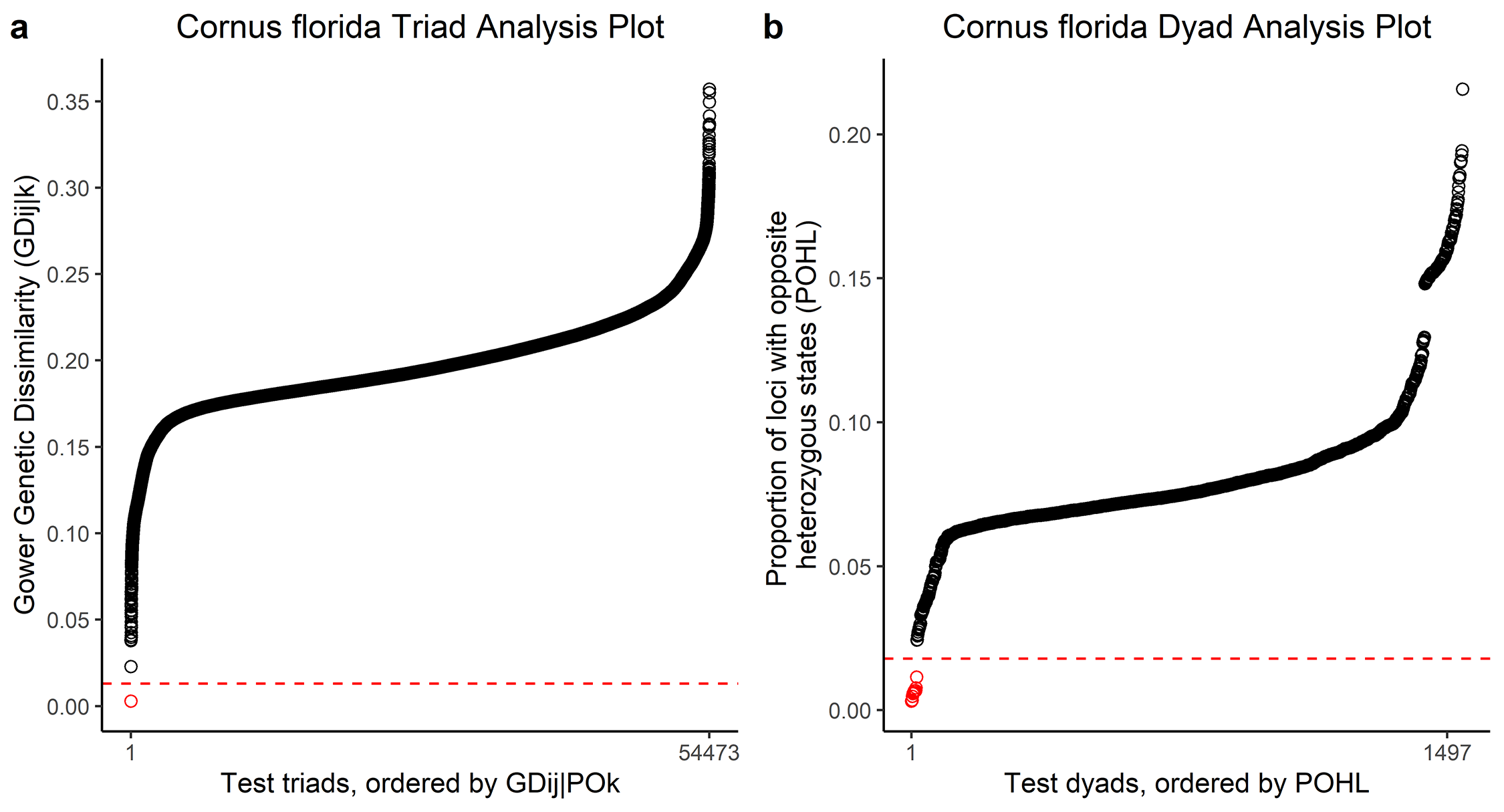

Supplement: S3 Fig — a) Triad analysis plot for test triads, ordered by Gower Dissimilarity. b) Dyad analysis plot for test dyads, ordered by POHL. Red dotted lines indicate the halfway point of each significant gap and red data points are the triads/dyads that were tested for significance with a second Dixon test. (TIF) [file pone.0307326.s003.tif]

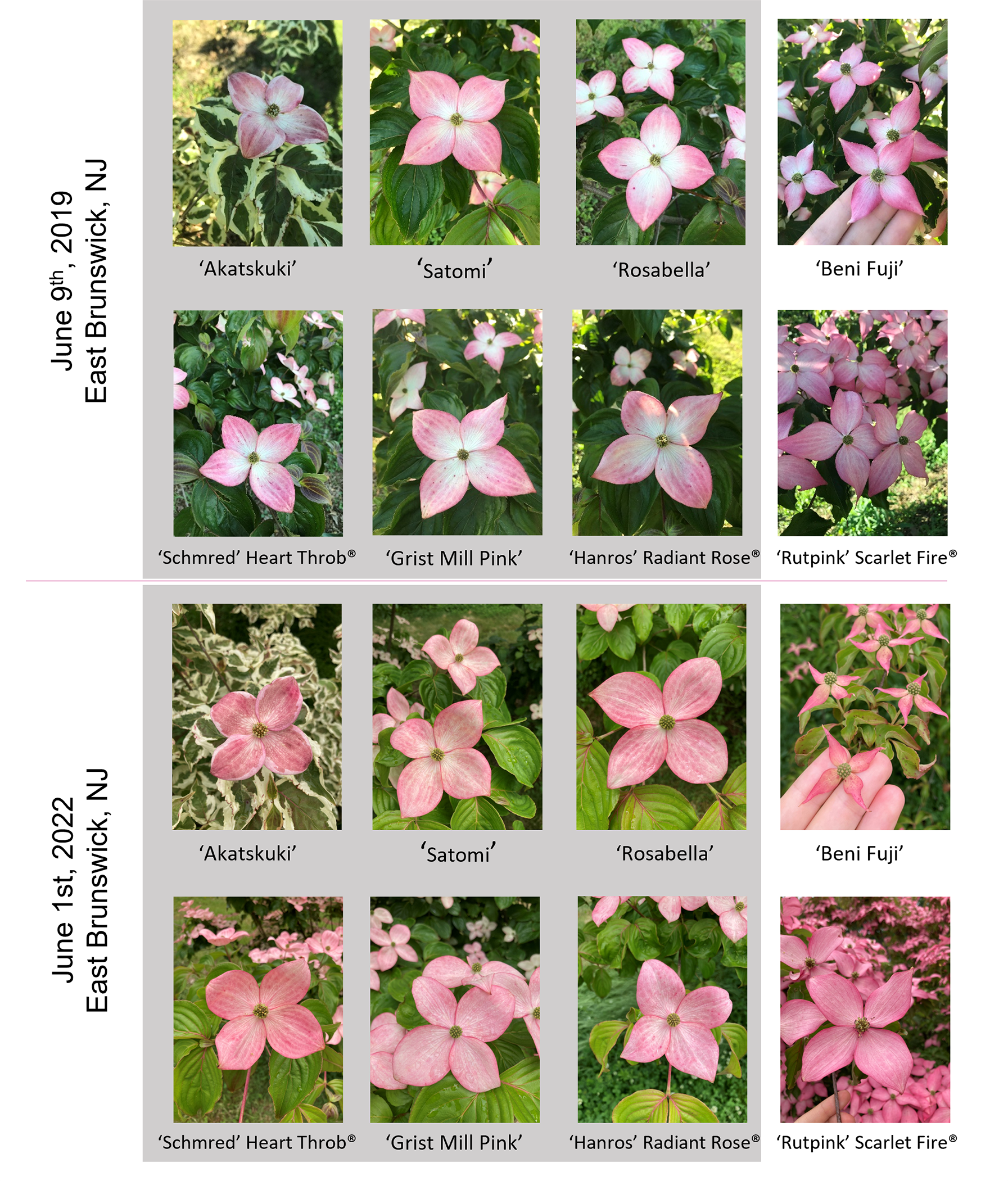

Supplement: S4 Fig — The location and date of photos is indicated to the left of each panel. The plants in the gray rectangles clustered together in the NJ tree made from 7,622 SNP and Indel markers and shared between 0.996 and 0.999 similarity based on a modified Gower’s similarity metric. Photos are not to scale. (TIF) [file pone.0307326.s004.tif]

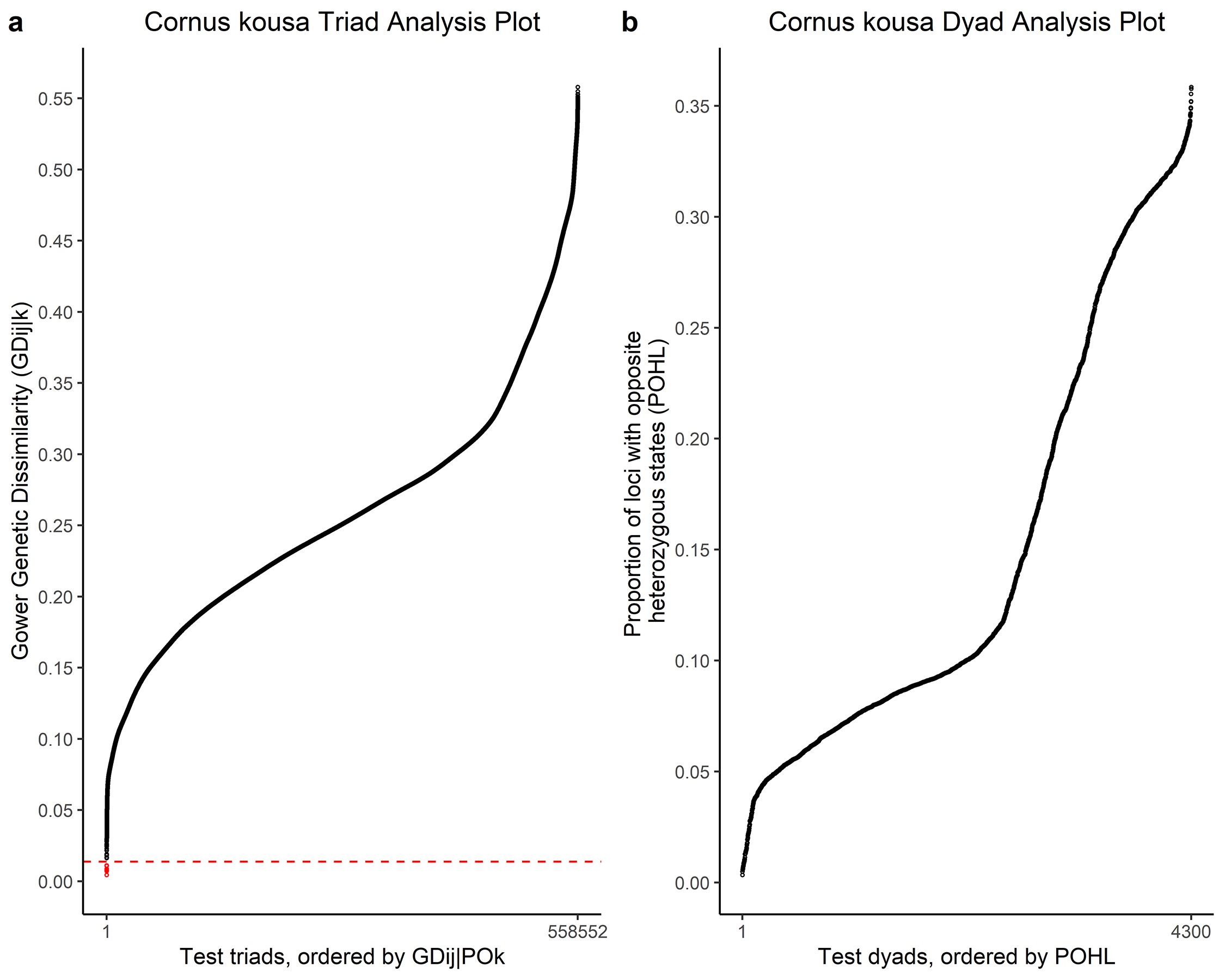

Supplement: S5 Fig — a) Triad analysis plot for test triads, ordered by Gower Dissimilarity. b) Dyad analysis plot for test dyads, ordered by POHL. Red dotted lines indicate the halfway point of each significant gap and red data points are the triads/dyads that were tested for significance with a second Dixon test. (TIF) [file pone.0307326.s005.tif]
