# Supplementary material for: Genetic diversity analysis of big-bracted dogwood (Cornus florida and C. kousa) cultivars, interspecific hybrids, and wild-collected accessions using RADseq
Source: PLoS One. 2024 Jul 25;19(7):e0307326. doi: 10.1371/journal.pone.0307326 (PMC11271954; doi:10.1371/journal.pone.0307326)
Supplement: S1 Text — Additional information about synonymous cultivars of C. florida and C. kousa, C. florida parent-progeny relationships, and hybrid analysis sibling groups. (DOCX) [file pone.0307326.s011.docx]

**Supplementary discussion**

***C. florida* cultivar discussion**

Other cultivars in the ‘Cherokee Chief’ clonal group along with ‘Cherokee Sunset’ and ‘Pumpkin Patch’ are 46823-H, ‘Grand Scarlet’, ‘Royal Red’, and ‘Prosser’ (**Fig 3**a). 46823-H is an unnamed, pink-bracted tree that was donated to the USNA in 1980. ‘Grand Scarlet’s only mention is as cultivar for sale at a rare and unusual cultivar nursery in Oregon. ‘Royal Red’ has previously been documented as phenotypically identical to ‘Cherokee Chief’ and likely represents a mixup in the trade (Cappiello and Shadow 2005). ‘Prosser’ is a synonym for ‘Prosser Red’, one of the earliest f. *rubra* cultivars, discovered in 1917. It has small pink bracts and is listed as the female parent of ‘Cherokee Chief’ and ‘Sweetwater Red’ (Cappiello and Shadow 2005).

‘Spring Song’, ‘Pink Flame’, and ‘Hess Select Red’ are also unexpectedly genetically identical (**Fig 3**). ‘Pink Flame’ is a faster growing green, yellow, and pink variegated form selected as a sport of the green, white, and pink variegated ‘Welchii’ (not sampled in this study) (Cappiello and Shadow 2005). ‘Spring Song’ and ‘Hess Select Red’ are both normal pink-bracted cultivars, however, ‘Spring Song’ is mentioned more in the literature. This genetic evidence suggests that ‘Spring Song’ or ‘Hess Select Red’ could be the origin of the ‘Welchii’ sport.

‘Cloud Nine’ and ‘Barton’s White’ (synonym ‘Barton’) were genetically identical, which was previously documented (Windham and Trigiano 1998). A trio of variegated cultivars, ‘First Lady’, ‘Golden Nugget’ and ‘Hohman’s Golden’, were also genetically identical. They have previously been noted as phenotypically similar with golden-yellow leaf margins and green centers (Witte, Windham et al. 2000). The three white, sterile double bracted *C. florida*, ‘Pluribracheata’, ‘Plena’, and ‘Eternal Dogwood’ have GSs of 1.000. ‘Pluribracheata’ is considered the original white double bracted form, originating from Orange County, NC in the early 1900’s. It displays six to eight or more bracts per bloom (Cappiello and Shadow 2005). ‘Plena’ is described as a catch-all designation for a variety of double-bracted forms (Cappiello and Shadow 2005).‘Eternal Dogwood’ is a selection described as having 12-20 bracts per flower, more than a typical dogwood (Simmen 2002). However, the ‘Pluribracteata’, ‘Plena’, and Eternal Dogwood’ in the Rutgers dogwood cultivar collection have indistinguishable inflorescences with 12-24 bracts per individual. Additional resampled ‘Pluribracteata’ from Bernheim and ‘Plena’ from USNA were also identical.

***C. florida* parentage analysis**

In contrast, there were a few possible or expected parent-progeny relationships that were not validated by the analysis. ‘Comco No. 1’ Cherokee Brave™ is supposedly a seedling of ‘Cherokee Chief’ (Ament, Windham et al. 2000, Cappiello and Shadow 2005), however this was not mentioned in the Cherokee Brave™ plant patent , and was not supported by this study’s data. The dyad was not significant in the dyad analysis with $P_{OHL}$=0.055, well above the gap-based threshold. ‘Super Princess’ was not the progeny of ‘Cherokee Princess’ ($P_{OHL}$= 0.069) as has been suggested by some nursery catalogs. H4AR15P25 is a PM disease resistant selection in the Rutgers breeding program with unknown parentage. In the NJ tree, it clusters strongly with ‘Cloud Nine’, an older cultivar that also shows moderate PM disease resistance and ‘White Cloud’ an old, heavily flowering cultivar with rounded bracts(Cappiello and Shadow 2005). However, the dyad analysis does not support either of these cultivars as parents of H4AR15P25 ($P_{OHL}$=0.030 and $P_{OHL}$=0.034, respectively), even though they are quite similar genetically.

***Cornus kousa* analysis**

‘Summer Majesty’ is a questionable accession because the clone from the USNA was subspecies chinensis and distinct from the Rutgers ‘Summer Majesty’ which is a hybrid. In addition, ‘China Girl’ from Rutgers was clonally identical to ‘Snowy Peak’ and ‘Highland’ yet a resampled ‘China Girl’ from the Bernheim arboretum was distinct, and on the borderline of ssp. hybrid. However, some of the ssp. chinenses cultivars were highly genetically similar (GS> 0.995) suggesting that they are clones. There were clonal groupings of ‘China Girl’, ‘Snowy Peak’, and ‘Highland’; ‘Big Dipper’ and ‘Galzam’ Galilean®; ‘Spiritual’ and ‘Samzan’ Samaritan®; ‘Emerald Star’ and ‘Ticknor’s Choice’; and ‘Greensleeves’ and ‘Snow Tower’. ‘Greensleeves’ and ‘Snow Tower’ were both resampled from two different sources, with the same results. ‘Snow Tower’ has likely been incorrectly labeled in the trade. It was originally described as an upright columnar cultivar but does not have this form in the Rutgers dogwood cultivar trial.

‘Milky Way’ was ssp. *chinensis* in our analysis, but it is important to note that the original ‘Milky Way’ cultivar was not a single clone. For ‘Milky Way’ propagation, scion wood was taken from a stock block of 15 seedlings from diverse seed sources at Wayside Gardens in the 1960s and 1970s (Orton 1991). Therefore depending on the origin, ‘Milky Way’ may not be ssp. *chinensis*.

‘Summer Fun’ and the initially sampled ‘Snowboy’ (Porter Collection) were genetically indistinguishable, however, a resampled ‘Snowboy’ from the Arnold Arboretum was ssp. *kousa*. This is the original ‘Snowboy’ as it was sourced via Maryland’s Brookside Gardens from the Japanese Sakata nursery, which selected the original cultivar.

**Hybrid analysis**

In the discussion to this point, most groups of OP siblings tested had similar genetic makeup, however, KF137-123, KF137-47, and KF137-62 were very different genetically from each other and their expected hybrid makeup, indicating that a mistake had occurred during labeling or propagation. These trees with parentage KF81-1 × *C. kousa* K2 were expected to be 12.5% florida in a kousa background, but instead KF137-123 was 42.8% florida, KF137-47 was 0% florida and 7.0% nuttallii, and KF137-62 was 0% florida and 3.2% nuttallii.

Advanced backcross hybrid status was not confirmed for *C.* x *rutgersensis* backcrossed to *C. kousa*. As discussed above, KF137-62 was unexpectedly 3.2% nuttallii, not florida, in the C. kousa background, indicating a possible germplasm mistake. Its three OP progeny H3DR03P75, H3DR04P03, and H3DR05P13 were mostly 100% kousa instead of the expected 6.25% florida and 93.75% kousa. (H3DR03P75 was 99.3% *C. kousa* and 0.7% *C. nuttallii*) The data does not exclude the possibility that H3DR03P75, H3DR04P03, and H3DR05P13 are the grand progeny of KF81-1, but instead raises the possibility that some reproductive barrier is favoring the loss of *C. florida* DNA in the *C. kousa* background. With the above information, it makes sense that H3DR04P03’s OP offspring H3G2R10P71 (RUT 19-38) and H3G2R10P73 (RUT 19-39), were also 100% kousa instead of 3.125% florida and 96.875% kousa.

Ament, M. H., M. T. Windham and R. N. Trigiano (2000). "Determination of parentage of flowering dogwood (*Cornus florida*) seedlings using DNA amplification fingerprinting." Journal of Arboriculture **26**(4): 206-212.

Cappiello, P. and D. Shadow (2005). Dogwoods: The Genus Cornus. Portland, Oregon, Timber Press Incorporated.

Nicholson, H. A. (1997). Dogwood Tree 'Comco No. 1'. United States Patent and Trademark Office, Commercial Nursery Co., Inc. **US PP10,166**.

Orton, E. R. (1991). *Cornus kousa* var. *chinensis* 'Milky Way' and Name Recognition in the Nursery Industry. Combined Proceedings of the International Plant Propagator's Society.

Simmen, P. W. (2002). Dogwood Tree Named 'Eternal Dogwood'. United States Plant Patent and Trademark Office, PW Simmen and C Domenig. **US PP13,069 P2**.

Windham, M. T. and R. N. Trigiano (1998). "Are ‘Barton’ and ‘Cloud 9’ the Same Cultivar of *Cornus florida* L.?" Journal of Environmental Horticulture **16**(3): 163-166.

Witte, W. T., M. T. Windham, A. S. Windham, F. A. Hale, D. C. Fare and W. K. Clatterbuck (2000). Dogwoods for American Gardens, The University of Tennessee Extension.
